# AgCu Bimetallic Electrocatalysts for the Reduction of Biomass-Derived Compounds

**DOI:** 10.1021/acsami.1c02896

**Published:** 2021-05-11

**Authors:** Giancosimo Sanghez de Luna, Phuoc H. Ho, Adriano Sacco, Simelys Hernández, Juan-Jesús Velasco-Vélez, Francesca Ospitali, Alessandro Paglianti, Stefania Albonetti, Giuseppe Fornasari, Patricia Benito

**Affiliations:** † Department of Industrial Chemistry “Toso Montanari”, Università di Bologna, Viale Risorgimento 4, 40136 Bologna, Italy; ‡ Center for Sustainable Future Technologies @POLITO, Istituto Italiano di Tecnologia, Via Livorno 60, 10144 Turin, Italy; § Department of Applied Science and Technology (DISAT), Politecnico di Torino, C.so Duca degli Abruzzi 24, 10129 Turin, Italy; ∥ Department of Civil, Chemical, Environmental and Materials Engineering, Università di Bologna, Via Terracini 28, 40131 Bologna, Italy; ⊥ Fritz-Haber-Institut der Max-Planck-Gesellschaft, Faradayweg 4-6, 14195 Berlin, Germany; # Department of Heterogeneous Reactions, Max Planck Institute for Chemical Energy Conversion, Mülheim an der Ruhr 45470, Germany

**Keywords:** Ag, Cu, electrocatalyst, foam, electroreduction, 5-hydroxymethylfurfural, 2,5-bis(hydroxymethyl)furan

## Abstract

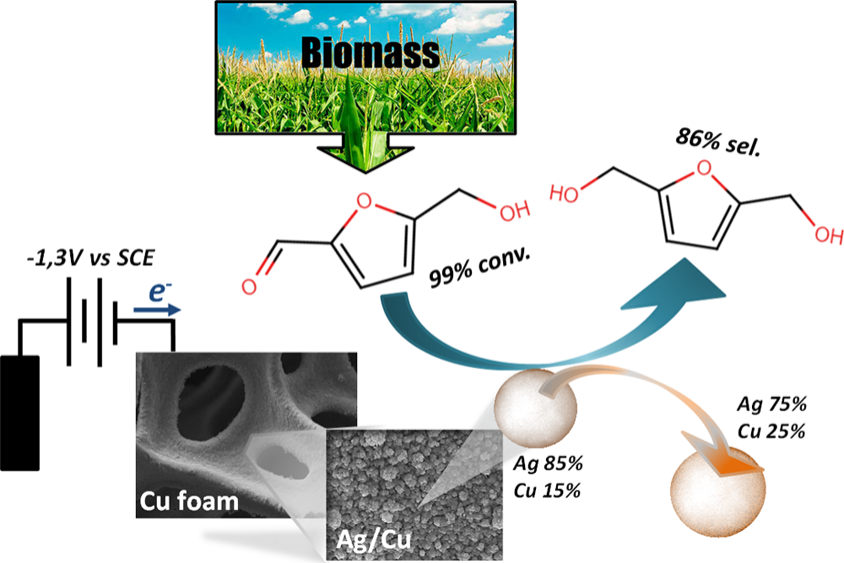

The electrochemical transformation of biomass-derived compounds (e.g., aldehyde electroreduction to alcohols) is gaining increasing interest due to the sustainability of this process that can be exploited to produce value-added products from biowastes and renewable electricity. In this framework, the electrochemical conversion of 5-hydroxymethylfurfural (HMF) to 2,5-bis(hydroxymethyl)furan (BHMF) is studied. Nanostructured Ag deposited on Cu is an active and selective electrocatalyst for the formation of BHMF in basic media. However, this catalyst deserves further research to elucidate the role of the morphology and size of the coated particles in its performance as well as the actual catalyst surface composition and its stability. Herein, Ag is coated on Cu open-cell foams by electrodeposition and galvanic displacement to generate different catalyst morphologies, deepening on the particle growth mechanism, and the samples are compared with bare Ag and Cu foams. The chemical–physical and electrochemical properties of the as-prepared and spent catalysts are correlated to the electroactivity in the HMF conversion and its selectivity toward the formation of BHMF during electroreduction. AgCu bimetallic nanoparticles or dendrites are formed on electrodeposited and displaced catalysts, respectively, whose surface is Cu-enriched along with electrochemical tests. Both types of bimetallic AgCu particles evidence a superior electroactive surface area as well as an enhanced charge and mass transfer in comparison with the bare Ag and Cu foams. These features together with a synergistic role between Ag and Cu superficial active sites could be related to the twofold enhanced selectivity of the Ag/Cu catalysts for the selective conversion of HMF to BHMF, that is, >80% selectivity and ∼ 100% conversion, and BHMF productivity values (0.206 and 0.280 mmol cm^–2^ h^–1^) ca. 1.5–3 times higher than those previously reported.

## Introduction

The electrocatalytic hydrogenation or reduction of biomass-derived compounds is a fully sustainable alternative to thermocatalytic hydrogenation processes and a path for the storage of renewable electric energy into chemicals and liquid organic fuels.^1−4^ The reduction of molecules containing aldehydes and aromatic or furan groups has been investigated. For instance, the electrochemical conversion of benzaldehyde to benzyl alcohol is a model reaction for the ambient temperature post-pyrolysis treatment of bio-oil.^5,6^ Moreover, the electroreductions of furfural^7,8^ and 5-hydroxymethylfurfural (HMF)^9−11^ biomass-platform molecules produce furfuryl alcohol and 2,5-bis(hydroxymethyl)furan (BHMF), which are precursors of polymers, and 2-methylfuran and 2,5-dimethylfuran (DMF), which can be used as fuel additives.

The electrochemical reduction of aldehydes has been reported to occur through either a coupled proton–electron transfer process^5^ or electrocatalytic hydrogenation with H_ads_ in situ formed by the Volmer reaction (since water is usually the solvent).^8^ Thus, two main competing reactions have been identified: (i) hydrodimerization leading to the formation of a diol, fostered at high substrate concentration, and (ii) hydrogen evolution reaction (HER), promoted at high overpotential.^12^

The reactivity of the aldehydes is determined by the structure of the molecule,^3,13^ e.g., the aromaticity in benzaldehyde and furfural favors the rate of carbonyl conversion. Moreover, the selectivity of the reduction is caused by an interplay between the type of electrocatalyst and reaction conditions (e.g., electrolyte pH and potential). The pH of the electrolyte controls the reaction intermediates and the electron and H^+^ transfer mechanism.^1^ On the other hand, base or noble metals drive the reaction pathway toward the electrochemical hydrogenation or dimerization.^5,6^ The binding energies of the carbonyl group and the metallic catalysts control the activity in the hydrogenation of benzaldehyde, according to the Sabatier principle.^14^ The intrinsic rate of this reaction is directly proportional to the average size of Pt particles deposited on carbon due to the ensemble size sensitivity of the adsorption of the aromatic molecules.^15^ Meanwhile, modifications in the carbon support (e.g., Brønsted Acid sites) tune the proton-coupled electron transfer.^16^

**Scheme 1:**
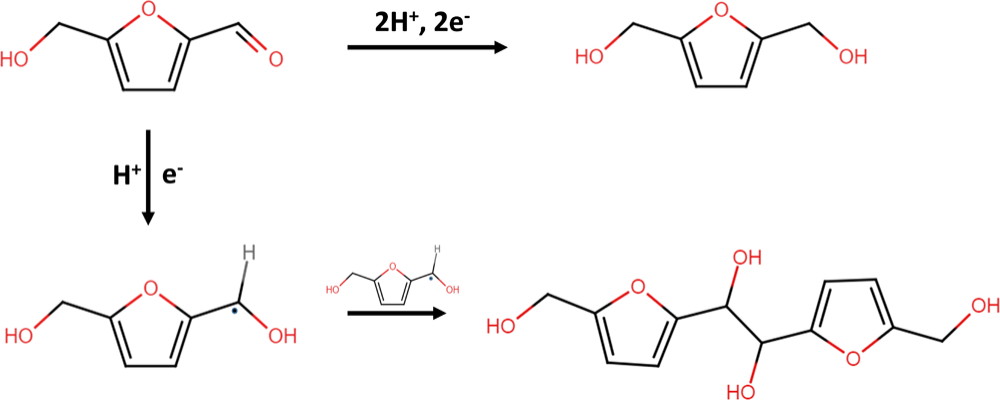
Electrochemical Conversion of HMF: Route for the Formation of BHMF (Top) and 5,5′-Bis(hydroxymethyl)hydrofuroin (Bottom)

In particular, the aldehyde group in the HMF molecule is readily converted to the corresponding alcohol, selectively producing BHMF at high Faradaic efficiency (FE).^9^ This occurs in a diluted electrolyte (0.02 M HMF) and basic media (borate buffer, pH = 9.2) over Ag-based catalysts (Scheme 1). Meanwhile, as the HMF concentration increases, a 1e^–^/1H^+^ transfer occurs, generating a radical that reacts with another radical, forming the diol (5,5′-bis(hydroxymethyl)hydrofuroin).^6,17^ The competition between HMF reduction to BHMF and hydrodimerization and HER is potential-dependent.^10^

Not only the reaction conditions but also the electrocatalyst properties, e.g., shape and composition, are of paramount importance. The role of Ag nanostructuring in the performance of the catalysts has been highlighted. Carbon-supported Ag nanoparticles have an intrinsically higher activity than bulk Ag.^10^ Moreover, Ag electrodes with a dendritic fractal morphology, deposited on Cu plates by galvanic displacement, show enhanced activity in comparison with flat electrodes obtained by sputtering.^9^ Recently, some of us reported that the combination of electrodeposited Ag nanoparticles and 3D Cu open-cell foams produces electrocatalysts with hierarchical porosity, outperforming flat electrodes.^11^ Nanostructuring is an efficient way to intensify the electrocatalyst activity since it provides a greater number of active sites compared to the bulk metal, increasing the exchanged current.^18^ Galvanic displacement^17,19^ and electrodeposition^20−22^ are two simple methods to nanostructure the surface of Cu supports with Ag particles of different morphologies. However, the changes in the morphology and subsequently on the electroactive surface area of Ag may modify the mass transfer and selectivity in an electrocatalytic process, as it has been shown for the CO_2_ electroreduction processes.^23,24^

Besides Ag nanostructuring, it would appear that the combination of both Ag and Cu in the electrocatalysts promotes the HMF reduction,^11,17^ a behavior also reported for O_2_^25,26^ and CO_2_ reduction reactions.^27,28^ In our previous work about Ag electrodeposited on Cu foams, Pb underpotential deposition (UPD) suggests that both Ag and Cu are electroactive,^11^ whilst Zhang et al. show that the surface of displaced catalysts is made of both Ag and Cu.^17^ However, the nature and structure of the active sites are not clear, hence hindering the understanding on the electrocatalytic performance.

The presence of Cu on the surface of displaced and electrodeposited Ag dendrites has been previously reported^22,29−31^ as well as the formation of Ag@Cu particles by galvanic replacement.^32^ However, it is challenging to characterize the materials to exclude the contribution of the support to the Ag dendrite composition or whether the presence of Cu comes from sample handling.^22^ At this point, it should be considered that Cu and Ag are immiscible at room temperature at almost all ratios,^33,34^ though the codeposition of Ag and Cu can be achieved by photodeposition^35^ and electrodeposition.^36−40^ Even in some cases, metastable alloys or non-equilibrated AgCu bimetallic particles, where the kinetics of the phase segregation are delayed, have been identified.^35,37^

Ag/Cu nanostructured catalysts show many advantages; however, they may also suffer from changes in the nanoparticle coatings during liquid phase electrolysis at cathodic potentials, such as sintering, detachment, or modification of the surface composition. Consequently, the electrocatalytic activity will be altered. However, these aspects have not been properly investigated neither during the electrocatalytic reduction of HMF nor for the reduction of other types of aldehydes.

The aim of this work is to fill the gaps in the field of nanostructured Ag/Cu electrocatalysts for the reduction of aldehydes in biomass-derived compounds to the corresponding alcohols, e.g., HMF to BHMF, which could be extended to the reduction of other biomass-derived molecules. In particular, not only are the morphology and size of the deposited particles (usually related to the efficiency of nanostructured electrocatalysts) investigated, but also efforts are devoted to elucidate the nature of the active species (composition and element distribution) and their stability during the electrochemical process. To achieve this aim, Ag coatings with different textural properties are deposited on Cu open-cell foams by electrodeposition and galvanic displacement. The so-obtained electrocatalysts and the particle growth mechanism are investigated and compared with Ag and Cu bare foams. The chemical–physical, electrical, and electrochemical properties of the catalysts are deeply characterized by a combination of X-ray (XRD and XPS) and microscopy (FE-SEM and HRTEM) techniques, micro-Raman, voltammetry, electrochemical impedance spectroscopy (EIS), and, for the first time, electrical resistance tomography (ERT). An attempt is made to establish the relationship among electrocatalyst structure, composition, and performance under a constant applied potential on the selective reduction of HMF to BHMF. The data obtained indicate that the formation of bimetallic AgCu particles promotes the selective production of BHMF and enhances the charge and mass transfer in comparison with bare Ag and Cu foams. However, the Cu enrichment of the bimetallic particles occurs during electrochemical tests, making rather challenging the identification of the real active sites. The dynamic interdependence of catalyst stability and activity under electrochemical tests highlights the relevance of investigating in situ the catalysts under reaction conditions, to track their evolution and set the basis for the development of advanced electrocatalysts for HMF electrocatalytic conversion and in general for the electrochemical biomass valorization. Thus, we plan to investigate the changes in the electronic structure and in the morphology of the catalyst during reaction conditions by combining in situ X-ray spectroscopy with in situ electron microscopy using some of our recently developed approaches.^41,42^

## Experimental Section

### Materials and Chemicals

Cu and Ag commercial foam panels were supplied by Alantum. Chemicals used in this work were sodium hydroxide (≥98%, Sigma-Aldrich), boric acid (≥99.5%, Sigma-Aldrich), silver nitrate (99.9%, Alfa Aesar), copper sulfate (99.95%, Sigma Aldrich), and 5-hydroxymethylfurfural (99%, AVA Biochem). 2,5-Bis(hydroxymethyl)furan (Toronto Research Chemicals) was used as a standard for high-performance liquid chromatography (HPLC) analysis. Lead nitrate (99.5%), sodium potassium tartrate (99%), and sulfuric acid (96%) (Sigma-Aldrich) were used for the determination of the electroactive surface area (EASA). All chemicals were used without further purification. Ultrapure water (UPW, 18 MΩ cm) was used for the preparation of all aqueous solutions.

### Preparation of Electrocatalysts

Cu and Ag electrodes of 10 mm × 10 mm were cut from the 1.6 mm-thick and 450 μm-cell-size foam panels (geometric surface area, 2.64 cm^2^). The pieces were cleaned by washing with 2-propanol, UPW, and 1 M HCl for 5 min to remove surface oxides and UPW to remove HCl. Ag/Cu electrodes were synthesized by electrodeposition (Ag/Cu ED) and galvanic displacement (Ag/Cu GD).

Ag electrodeposition on Cu foams was performed in a single-compartment three-electrode cell controlled by a potentiostat/galvanostat Metrohm Autolab PGSTAT204, equipped with NOVA software. Foams were the working electrodes (WE), while a saturated calomel electrode (SCE) and a Pt wire were the reference electrode (RE) and counter electrode (CE), respectively. The RE was placed close to the surface of the WE in the center of the cell, while the CE was placed around them close to the walls of the cell. Electrodeposition was performed by applying a 25 s pulse at −0.9 V vs SCE (−0.24 V vs RHE), and the foam to be deposited was immersed in 25 mL of 5.0 mM AgNO_3_ aqueous solution electrolyte under magnetic stirring at 500 rpm.

Ag galvanic displacement was performed by immersing Cu foams in a beaker containing 25 mL of 5.0 mM AgNO_3_ aqueous solution for 5 min. The foams were attached to a rotor and kept under mechanical stirring of 200 rpm.

After synthesis, all the electrocatalysts were rinsed gently with ethanol and water. The selection of the deposition conditions was performed as preliminary work. In the Supporting Information (Tables S1 and S2), selected samples are reported, which also allows us to follow the particle growth mechanism.

For comparison purposes, nanostructured Ag/Ag and Cu/Cu electrodes were synthesized by electrodeposition on Ag and Cu foams, respectively. Ag particles were electrodeposited on Ag foam by using a 5 mM AgNO_3_ aqueous solution and applying a cathodic pulse of −1.1 V vs SCE (−0.44 V vs RHE) for 50 s. The electrodeposition of Cu particles was carried out in an electrolyte containing 0.024 M CuSO_4_ and 0.08 M H_2_SO_4_ solution at −0.3 V vs SCE (0.006 V vs RHE) for 300 s.

### Characterization Techniques

Inductively coupled plasma-atomic emission spectroscopy (ICP-AES) analyses were performed using an Agilent Technologies 4210MP-AES instrument. The Ag amount loaded on the electrocatalysts was calculated by the difference between the Ag content in the solution before and after deposition. These solutions were diluted to fall within the range of the calibration curve (0–15 ppm), which was built starting from a 1000 ppm Ag standard in 5% HNO_3_. The emissions at 328.1 nm for Ag and 324.7 nm for Cu were evaluated. Moreover, the analysis of the solutions after HMF electrolysis and Ag deposition was carried out to identify (not quantify) Ag and Cu.

The X-ray diffraction (XRD) analysis was carried out directly at the foam specimens using a PANalytical X’Pert diffractometer equipped with a copper anode (λ_mean_ = 0.15418 nm) and a fast X’Celerator detector. Wide-angle diffractogram was collected over a 2θ range from 3 to 80° with a step size of 0.067° and counting time per step 60.95 s.

The surface morphology of the foam electrodes was examined by scanning electron microscopy/energy-dispersive spectroscopy (SEM/EDS) and field emission scanning electron microscopy/energy-dispersive spectroscopy (FE-SEM/EDS). The SEM was an EP EVO 50 Series Instrument (EVO ZEISS) equipped with INCA X-act Penta FET Precision EDS microanalysis and INCA Microanalysis Suite Software (Oxford Instruments Analytical). The accelerating voltage was 20 kV, and the spectra were collected for 60 s. The FE-SEM was a ZEISS Leo 1530 equipped with INCA EDS microanalysis and INCA Microanalysis Suite Software (Oxford Instruments Analytical). The accelerating voltage was 10 kV, and the EDS spectra were collected during a period of 60 s.

Transmission electron microscopy characterization was carried out using a TEM/STEM FEI TECNAI F20 microscope, equipped with an EDS analyzer. The coating was removed by scratching the foam surface, and then it was suspended in ethanol under ultrasounds for 2 h. The suspension was subsequently deposited on a holey carbon film supported by a Au grid and dried at 100 °C before doing the measurement at 200 keV. Particle size distribution was processed considering around 300 particles.

Micro-raman spectra were obtained using a Renishaw Raman Invia spectrometer configured with a Leica DMLM microscope. An Ar^+^ laser source (λ = 514.5 nm, *P*_out_ = 30 mW, considering the decrease in power due to the plasma filter) was employed, setting the laser power by 10% of the source and accumulating four individual spectra for each measurement with an acquisition time of 10 s.

X-ray photoelectron spectroscopy (XPS) analysis was performed at the ISISS beamline of BESSY II in Berlin (Germany). In this facility, the photons are sourced from a bending magnet (D41) and a plane grating monochromator yielding an energy range from 80 to 2000 eV (soft X-ray range), a flux of 6 × 10^10^ photons/s with a 0.1 A ring current using a 111 μm slit, and an 80 μm × 200 μm beamspot size. The spectra were fitted using CasaXPS software.

The probe for electrical resistance tomography consists of 16 circular measurement electrodes, made of stainless steel with a diameter equal to 2 mm, set on a pipe with an inner diameter equal to 20 mm. The tested electrode was immersed in a solution of demineralized water and NaCl. The NaCl concentration was equal to 0.09 M. The local conductivity was measured with the ITS 2000 ERT instrumentation (Industrial Tomography Systems Ltd). The measurement electrodes were connected to the data acquisition system by coaxial cables. The measurements were based on the so-called circular adjacent strategy, in which electric current injected from adjacent electrodes pair at a time and the voltage difference is measured from the remaining pairs of electrodes. The procedure is repeated for all the independent pairs of electrodes. As for the reconstruction method for obtaining the conductivity maps from the electric potential measurements, the linearized (non-iterative) modified sensitivity back projection algorithm was selected,^43^ as implemented in the ITS System p2+ V8 software. The local conductivity on the measurement plane was obtained on a mesh of 1 mm × 1 mm. The number of local measurements inside a circular tomogram, *n*, was thus equal to 316. The amplitude and the frequency of the injected current were set at 15 mA and 9600 Hz, respectively, after preliminary calibration tests. For each set of acquisitions, 600 total instantaneous measurements were collected at a frequency of 0.92 frames per second. In the following, the dimensionless local conductivity (*X_i_*) that is computed as the ratio between the local conductivity (γ*_i_*) measured with the tested electrode set between the measurement electrodes and a reference conductivity (γ_*i*_^ref^) measured before the introduction of the tested electrode between the measurement electrodes will be analyzed. Values greater than unity mean that the conductivity of the medium has been increased because of the presence of the tested electrode. The tests were conducted under ambient conditions (25 °C, 1 atm).

### Electrochemical Measurements

Electrochemical measurements were controlled by a potentiostat/galvanostat Metrohm Autolab PGSTAT204, equipped with NOVA software; Cu or Pt wires were attached to the electrodes to enable connection to the potentiostat.

A three-electrode three-compartment cell, separated by glass frits, was used to perform all the electrochemical measurements. Working electrodes were Ag and Cu bare and coated foams, placed in the central compartment, with the reference electrode (SCE) put in electrolytic contact via a Luggin capillary. Counter electrodes were Pt wires, placed in the side compartments. The catholytes were 25 mL of 0.5 M borate buffer aqueous solution (pH 9.2) with and without HMF, and the anolyte was a 0.5 M borate buffer solution (pH 9.2) with 0.5 M sodium sulfite.

All potentials were reported vs SCE and RHE (V vs RHE = V vs SCE + 0.244 V + 0.0591 × pH). The cell was thermostatted with a water bath at 25 °C. For the determination of *R*_u_, the current interrupt method was used, applying a potential pulse of −1 mV for 2 ms. The i*R*_u_ drop for all the linear sweep voltammetries (LSVs) was compensated after measurements, whereas the constant-potential electroreductions were measured without compensation.

To avoid the presence of dissolved oxygen, all the solutions were purged with N_2_ before each electrochemical experiment, and a N_2_ flow was kept in the open space of the cell during experiments.

LSVs were recorded in a 0.5 M borate buffer solution (pH 9.2) with and without HMF for the electrochemical characterization of the catalysts. The potential was scanned from 0 to −1.4 V vs SCE (from 0.79 to −0.61 vs RHE) at scan rates of 1 mV s^–1^ without HMF and 5 mV s^–1^ with HMF. The slowest scan rate in the LSV without HMF was chosen to ensure the reduction of the electrocatalyst (see below).

The electroactive surface area (EASA) was estimated using Pb underpotential deposition (Pb UPD). The deposition/dissolution charge of Pb on the foams was compared with the response of polished Ag foil.^11^ Cyclic voltammetries (CVs) were recorded in a single compartment three-electrode cell between 0 and −0.6 V vs SCE (from 0.79 to 0.19 vs RHE) with a scan rate of 50 mV s^–1^. The electrolyte was an aqueous solution of 0.10 M sodium potassium tartrate containing 0.01 M H_2_SO_4_ and 3 × 10^–4^ M Pb(NO_3_)_2_.

Electrocatalytic reductions were performed potentiostatically at a cathodic potential of −1.30 vs SCE (−0.51 vs RHE) in deaerated HMF solution, flushing N_2_ in the overhead of the compartment where the WE was placed. The solution was kept under stirring with a magnetic bar at 1000 rpm.

The catalysts were first tested using a 0.02 M HMF solution and then with a 0.05 M HMF solution. The catalytic cycle started with a sequence of LSV in borate and borate plus HMF followed by electrolysis at a constant potential, and then the first two LSVs were repeated. At the end of the tests, the catalytic cycle was repeated by modifying or not the concentration of HMF (see Scheme S1). The LSVs performed before the HMF electroreduction ensured the reduction of any surface oxide species, therefore minimizing Faradaic loss during the process; conversely, those performed after the catalytic test allow checking for any change in the electrocatalysts after the reaction.

The reactions were carried out under total HMF conversion conditions, which were obtained through the transfer of the charge necessary to convert all HMF in solution into BHMF (i.e., through a 2e^–^ process), assuming a 100% faradic Efficiency. At the end, the solutions were collected and analyzed with HPLC. The reaction time depends on the type of catalyst and the reaction cycle; information about the length of the electrocatalytic tests can be found in the Supporting Information. The geometric surface areas of the electrodes were considered for calculating current densities.

Electrochemical impedance spectroscopy (EIS) was conducted in the same three-electrode three-compartment cell with a Biologic VSP-300 multichannel bipotentiostat. EIS experiments were performed in a borate plus 0.05 M HMF solution from 100 kHz to 100 mHz with an amplitude of 20 mV at a fixed −1.1 V vs SCE (−0.31 V vs RHE) potential.

### Product Analysis

To analyze the reaction solutions and to assess the product concentrations in the electrolytes, an HPLC Agilent 1260 Infinity Series equipped with a Cortecs T3 2.4 μm (4.6 × 100 mm) was used. The instrument operates at 30 °C with an autosampler (injection volume, 1 μL) and a diode-array detector set at 284 nm for the identification of HMF and 223 nm for the identification of BHMF. The analyses were performed with gradient elution in three steps: isocratic conditions for 6 min, with eluent composed of CH_3_CN/H_2_O 10/90 v/v ratio; gradient elution for 5 min until a CH_3_CN/H_2_O 50/50 elution ratio was obtained; and gradient elution for 4 min until a CH_3_CN/H_2_O 70/30 elution ratio was obtained. The flow rate was 0.7 mL min^–1^.

Conversion, selectivity, Faradaic efficiency (FE), BHMF productivity, and specific BHMF productivity were calculated with the following equations:
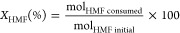

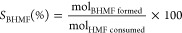

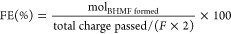
where *F* is the Faraday constant.
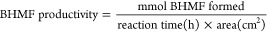

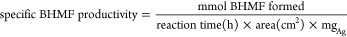


The area corresponds to the geometric area of electrodes (2.64 cm^2^). Due to the modifications in the EASA during the reaction, the specific BHMF productivity is normalized to the mg_Ag_ in the electrocatalyst.

For Ag/Cu ED and GD samples, average data is reported in the manuscript. Average values and standard deviations were calculated on three replicates obtained with different samples.

In the HPLC chromatograms, an intense peak close to the one for BHMF was detected, which was related to 5,5′-bis(hydroxymethyl)hydrofuroin, in agreement with gas chromatography–mass spectrometry (GC–MS) and electrospray ionization-mass spectrometry (ESI-MS) analyses.^11^ Unfortunately, we were not able to quantify the amount of the byproduct; hence, the area of the peak was used to estimate its formation during the electroreduction experiments over the different investigated electrocatalysts.

## Results and Discussion

### Characterization of the Electrocatalysts

**Figure 1:**
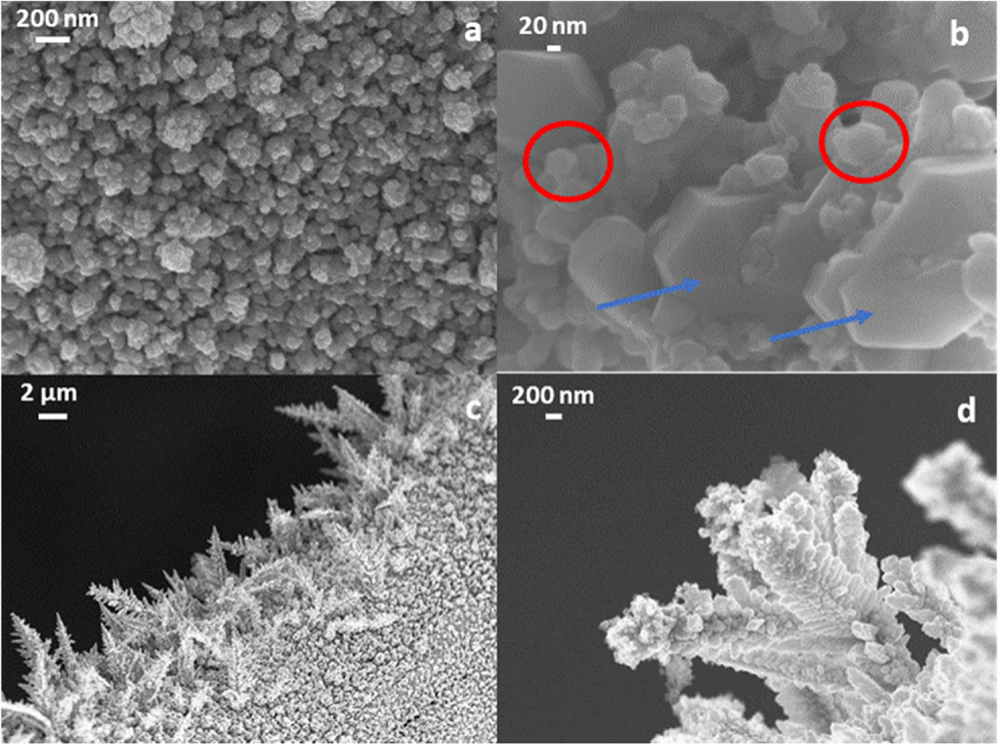
FE-SEM images of the Ag/Cu ED sample showing the deposition of agglomerates of nanoparticles (a, b) and dendrites (c, d). Red circles: octahedral particles; blue arrows: nanoplates.

In the electrodeposited sample (Figure 1), Ag/Cu ED, arrays of Ag particles coat the surface of the Cu foam (Figure 1a), while few dendrites develop in the edges of some struts (Figure 1c,d). The arrays are made of ill-defined 50–200 nm particles, well-faceted two-dimensional nanoplates, and some three-dimensional octahedral particles (20–50 nm) (Figure 1b). A cross section of the coating reveals a thickness of 300–600 nm (Figure S1). Stirring of the electrolyte during electrodeposition replenishes the solution in the electrode–electrolyte interface over the whole foam disk, resulting in a homogeneous coating of both the outer and inner surfaces of the foam disks (Figure S2).

**Figure 2:**
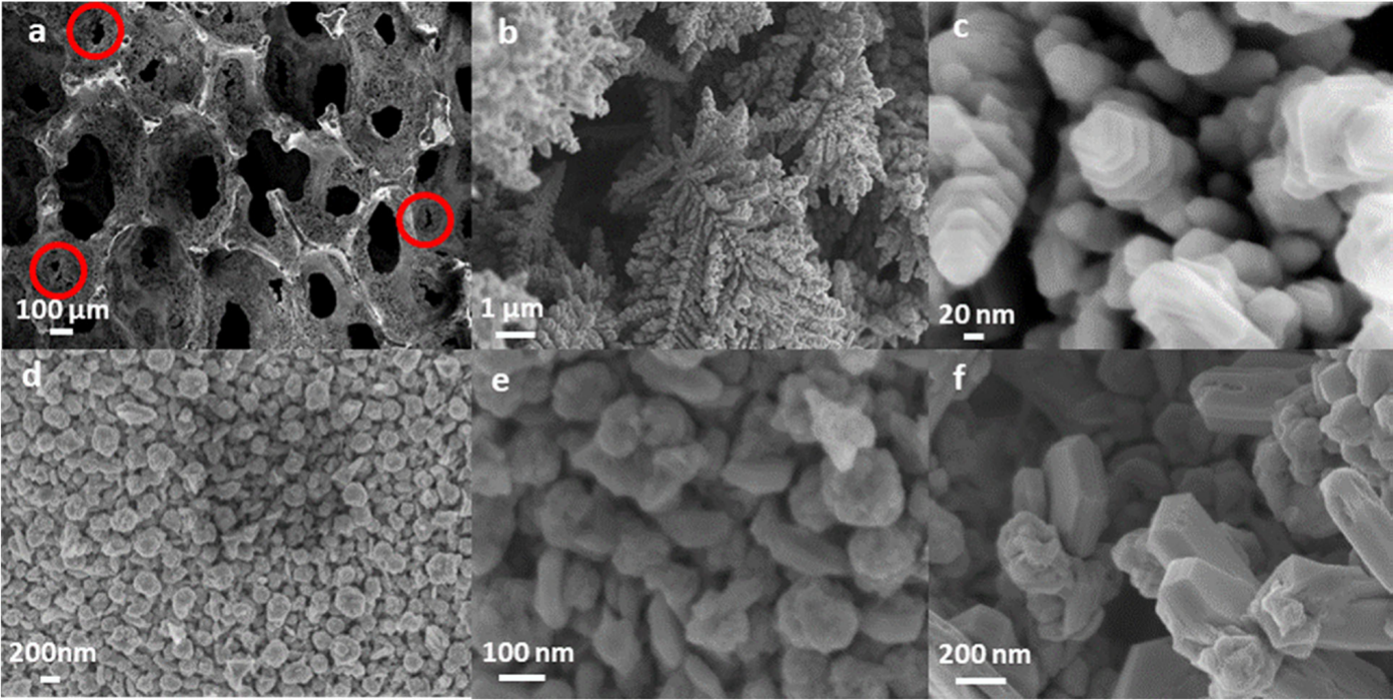
FE-SEM images of the Ag/Cu GD sample showing pores blocked or partially blocked with red circles (a), dendrites (b, c), and aggregates of nanoparticles at different magnifications (d–f).

In galvanic displaced samples (Figure 2), Ag/Cu GD, the formation of dendrites is largely enhanced in comparison with the electrodeposited sample. These dendrites grow on both the connections between struts and on the pore edges and partially block some pores (Figure 2a). Nonetheless, a homogeneous coating, made of agglomerates (ca. 100–350 nm) of well-faceted hexagonal particles and ill-defined smaller particles, is also observed on the surface of the struts and their connections (Figure 2d–f). The highly branched dendrites (Figure 2b), which have an overall length from 10 to 40 μm, are composed of hexagonal and ill-shaped motifs of ca. 120–200 and 50–100 nm, respectively, while more rounded particles, which are less than 50 nm, are on the top of the branches (Figure 2c). Note that the galvanic displacement is also performed under stirring, ensuring the coating of the whole foam surface.

**Figure 3:**
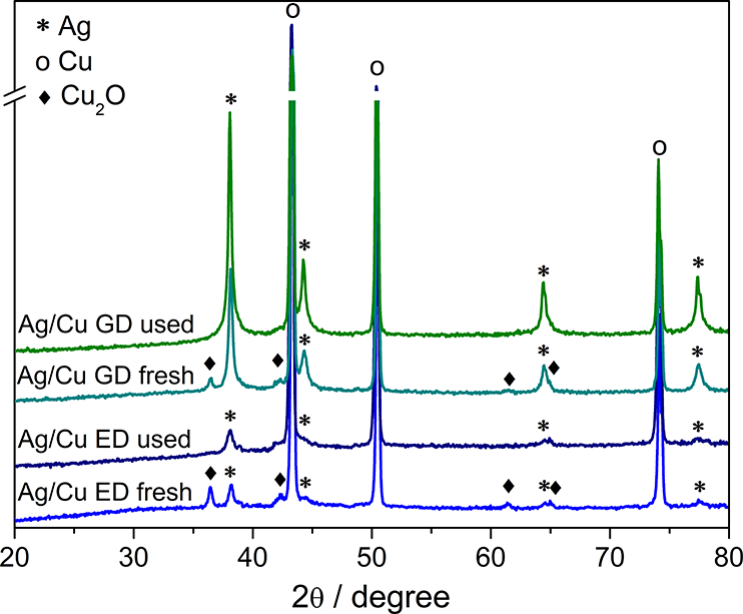
XRD patterns of fresh and spent Ag/Cu ED and Ag/Cu GD catalysts.

XRD patterns in Figure 3 confirm the deposition of cubic Ag^0^, growing preferentially in the (111) direction,^44^ for both electrodeposited and displaced samples. Nevertheless, more intense Ag^0^ reflections and a higher ratio of intensity between planes (111) and (220) are observed for Ag/Cu GD (i.e., *I*_(111)_/*I*_(220)_ = 6 and 3 for GD and ED samples, respectively) due to the larger Ag loading (three to five times greater) and the formation of dendrites. The development of Cu_2_O in both types of catalysts is evidenced by XRD and Raman in Figure 3 and Figure S3. In both preparation methods, ICP analysis of the solutions after the depositions reveals traces of copper.

**Figure 4:**
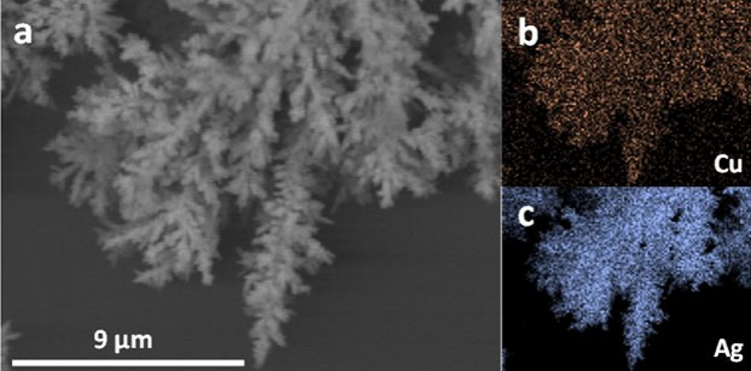
SEM image of a dendrite in the Ag/Cu GD sample (a) and Cu (b) and Ag (c) EDS elemental maps.

**Figure 5:**
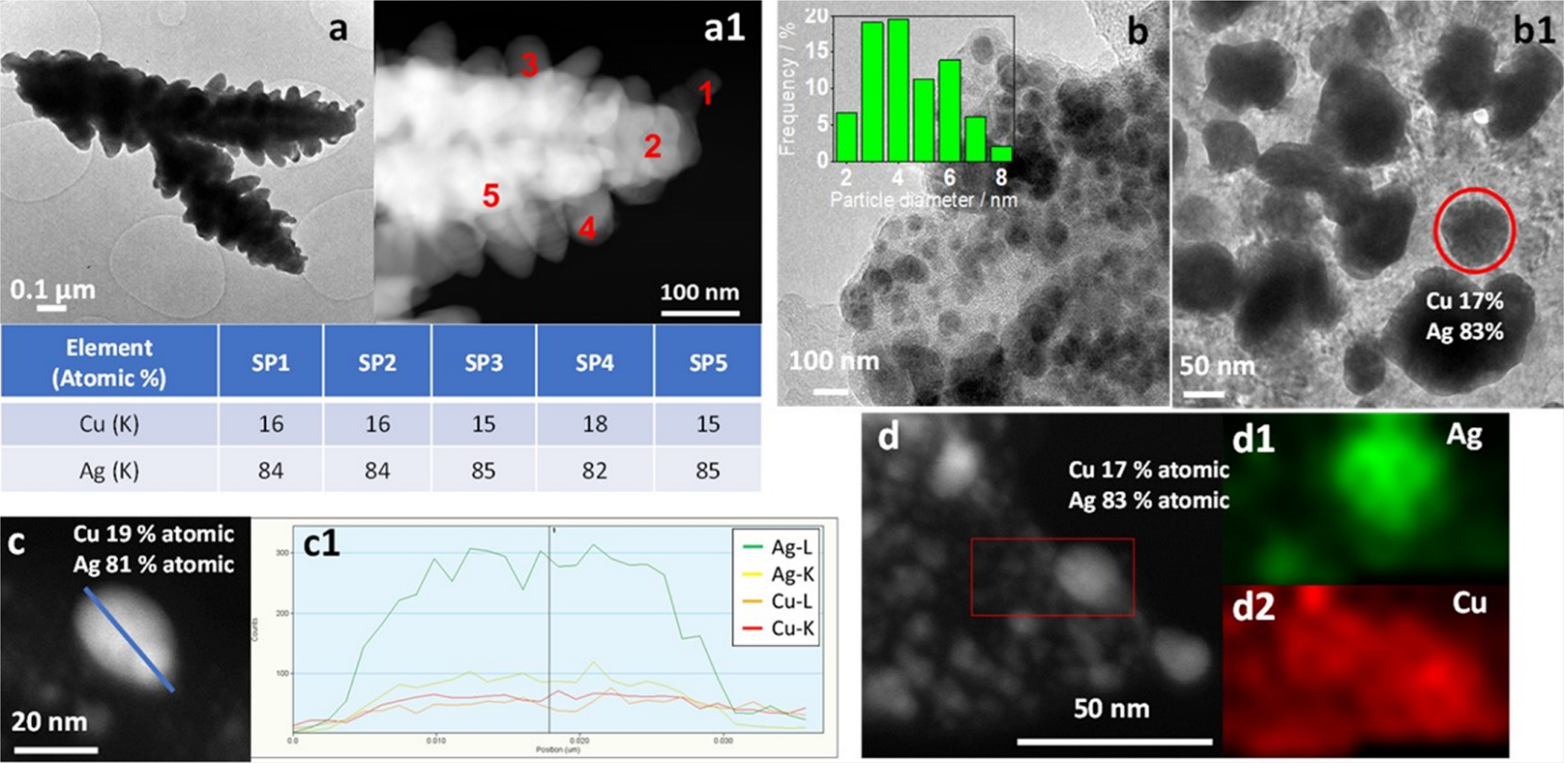
TEM characterization of Ag/Cu ED. HRTEM and HAADF/STEM images of a dendrite (a, a1). EDS data in the regions in (a1) are shown in the table. HRTEM images of the arrays of particle coating (b, b1). The particle size distribution of the small nanoparticles (inset in b). HAADF/STEM image of a Ag/Cu particle (c). The Ag and Cu elemental composition along the line is shown in (c1). HAADF/STEM image (d) and elemental maps of Ag (d1) and Cu (d2) on the region within the red square.

EDS elemental maps of dendrites in as-prepared electrodeposited and displaced samples indicate the coexistence of well-dispersed Ag and Cu elements (Figure 4 and Figure S4), though the formation of the AgCu alloy is ruled out by XRD (see also XPS data below). To further investigate the morphology and composition of the particles, the coatings were detached from the foam supports and were analyzed by HRTEM. The images and EDS data confirm that the dendrites in electrodeposited and displaced samples are made of interconnected and stacked particles containing both Ag and Cu (Figure 5a,a1 and Figure S5). Remarkably, the Ag/Cu atomic ratio is rather constant all over the dendrites with estimated values of 84-85/16-15 (Figure 5a1 and inset table and Figure S5). Interestingly, the analysis of the coatings detached from the flat zones of the foams reveals that besides the poorly defined and large arrays of particles observed by FE-SEM, small nanoparticles (2–7 nm for Ag/Cu ED) are well dispersed on the Cu oxidized and metallic support (Figure 5b,b1,d–d2 and Figure S6). Again, EDS analysis in an isolated particle (not supported) confirms the formation of AgCu bimetallic particles (Figure 5c,c1), with a composition similar to that of the dendrites. Note that both electrodeposited and galvanic displaced samples show similar types of particles, though these are bigger for the latter.

The Ag/Cu atomic ratio values herein obtained (ca. 85/15) are similar to those previously reported for AgCu catalysts prepared by electrodeposition^25,28,36^ and galvanic displacement^17^ as well as by melting in a microwave inductive furnace.^45^ In those works, some contrasting information can be found about the structure of the bimetallic particles. Choi et al. did not observe any shift in the diffraction peak positions of both Cu and Ag, thereby indicating that the alloy is not formed.^28^ On the other hand, Jin et al. stated the formation of the alloy in electrodeposited AgCu catalysts.^25^

**Figure 6:**
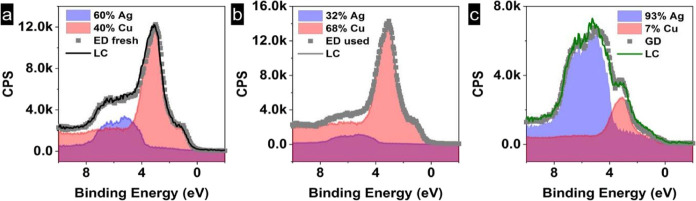
Valence band measurements of the Cu 3d and Ag 4d of the fresh Ag/Cu ED sample before reaction (a), Ag/Cu ED sample after reaction (b), and Ag/Cu GD sample (c).

In this work, XPS measurements confirm the information obtained by XRD and Raman. Ag is in the metallic phase, which is stable during handling, while both Cu metallic and Cu oxides are present on the surface of the electrodes. The Cu 2p and Ag 3d spectra of the electrocatalysts are shown in Figure S7. Some differences are found on the surface composition depending on the preparation method; the surface of the electrodeposited sample is enriched with Cu, while for the galvanic displacement, the dominant is a Ag-enriched surface. Valence band measurements reveal that the Ag 4d and Cu 3d components in electrodeposited and displaced samples can be fitted by a linear combination of their principal components (Ag and Cu reference samples), indicating that there is no alloy formation, i.e., lack of charge transfer between both components or distortion in the spectrum shape (see Figure 6).

The characterization results reveal that AgCu bimetallic particles are obtained by both ED and GD; however, the particle morphology and distribution depend on the type of Ag coating procedure. The particle morphology is an interplay between the reaction rate and mass transfer (mainly diffusion), which determines the chemical distribution on the growth front of particles.^46−48^ The reaction rate and mass transfer are modified not only by the concentration of AgNO_3_ and deposition time (and/or applied potential) but also by the location in the foam surface, as evidenced in the samples prepared under different deposition conditions (Tables S1 and S2).

At the early stage of the galvanic displacement, the concentrations of Ag^+^ in the solution–foam interface and bulk are similar. The large Cu area available for the displacement fosters the fast precipitation of Ag particles on all the foam surface, similar to those previously reported in the displacement of Cu with 1 and 5 mM AgNO_3_ solutions.^49,50^ The formation of particles randomly distributed that aggregate (Figure S8) indicates a high reaction rate and the tendency of the particles to decrease their surface energy. For instance, some platelets are actually composed of multiple hexagonal particles sharing the planar surfaces (Figure S8b). Afterward, the depletion of Ag^+^ in the vicinity of the foam surface may generate a concentration gradient. The diffusion-limited growth generates dendritic structures mainly on the most exposed areas of the foam, e.g., pore edges and tips of the struts. Galvanic displacement is faster on those higher surface energy zones, wherein the concentration gradient could be also higher. The formation of dendrites is governed by non-equilibrium and anisotropic conditions (kinetic regime); it may be explained by considering Mullins-Sekerka instabilities^51^ and the oriented attachment of particles.^52^ Indeed, well-defined hexagonal particles that share a common crystallographic orientation and bond to form a planar interface to reduce the surface energy are precipitated. The spontaneous self-organization of the hexagonal nanoparticles generates, in the initial steps, particle arrangements that grow perpendicular to the foam, as it can be observed in Figure S8a; subsequently, branching occurs since each corner of a motif crystal has the potential to develop a branch.^46^ As the deposition is further lengthened, the Ag deposition is more likely on the dendrites than on the foam substrate. This growth mechanism is validated by the characterization of samples prepared at different times. By halving the displacement duration (2.5 min), the formation of dendrites is largely suppressed, while the coating on the bulk foam surface is rather similar (Figure S9). On the other hand, by repeating twice the galvanic displacement (5 + 5 min), the rather uncoated foam struts can be distinguished perfectly in the SEM image in Figure S10, but the pores are filled by dendrites. Similar behavior occurs with a twofold concentrated AgNO_3_ solution (10 mM), Figure S11, though the solid loading deposited increases. The presence of Ag in the internal surface of the foam indicates that mass transfer inside the pores is not limited as reported by Lee et al.,^53^ a fact that could be related to the stirring of the solution. However, the forced convection may also modify the chemical gradient on the growth front and therefore the growth of the particles. To investigate the influence of the stirring on the morphology of the coatings, a sample was prepared under static conditions (Figure S12). The coating is unevenly distributed, decreasing the Ag loading deposited and the length of the dendrites.

The above discussion explains the morphology and distribution of the particles deposited during the displacement; however, EDS elemental maps suggest that the deposition is more complex than the sole formation of Ag particles of different morphologies. In galvanic displacement, Cu^0^ serves as the anode and it is oxidized to Cu ions (Cu^2+^ or Cu^+^) while Ag ions (Ag^+^) from the solution are reduced to Ag^0^ and preferentially deposited on the tips of the cathodic Ag seeds.^54^ Note that the electron transfer from Cu^0^ to Ag^+^ that is not in direct contact may be possible by electrical conduction through the Ag deposited.^55^ Copper oxidized species remain in the electrocatalyst, as evidenced by XRD and Raman; moreover, some of them are dissolved as confirmed by the presence of Cu^2+^ in the solution after displacement. Cu^2+^ cations are subsequentially reduced and simultaneously deposited with Ag, producing AgCu bimetallic particles.^30^

In the electrodeposition, the high driving force for Ag^+^ reduction fosters the generation of nuclei and small clusters. However, besides the chemical concentration gradients and the oriented attachment,^56^ the role of the spontaneous displacement and the distribution of the electrical field, governed by the applied potential, in the 3D support should be considered. At the overpotential applied (−0.9 V vs SCE), the particles do not grow directly over the bare foam but over an ultrathin layer of Ag deposits. This film is formed by spontaneous galvanic displacement within the time between the immersion of the foam in the electrolyte and the application of the cathodic pulse, i.e., 3 s (Figure S13). The precipitation in the most exposed areas may be fostered by an increased electrical field.^57^ However, the short pulse time (25 s) decreases the Ag^+^ concentration gradients and the dendrite formation thereof. An even shorter cathodic pulse (15 s) almost suppresses the deposition of dendrites, while the coating film on the foam surface is not largely modified (Figure S14). The same behavior is observed at a less cathodic potential (−0.7 V) for 25 s (Figure S15); the rate of electrodeposition decreases, the diffusion-limited conditions are not reached at a short time, and consequently, the dendrites do not develop. The opposite behavior occurs by prolonging the deposition time to 50 s at −0.9 V vs SCE, and it is possible to observe dendrites blocking the pores (Figure S16). Large amounts of Ag deposits could be also attained by electrodeposition, like for galvanic displacement, e.g., with a 10 mM AgNO_3_ solution at −0.9 V for 50 s (Figure S17). However, the aim of this work is not to achieve similar coverages by galvanic displacement and electrodeposition. Last, the stirring of the electrolyte is important; an inhomogeneous coating, with uncoated areas and bigger particles, is obtained under static electrodeposition conditions (Figure S18).

Similar to the displacement, the formation of AgCu bimetallic particles occurs as a side reaction during Ag deposition. The basic medium generated close to the foam surface by electroreduction of nitrate provokes the Cu foam oxidation and the Cu dissolution. The reprecipitation of copper is promoted by the cathodic reduction potential. This phenomenon is likely time-dependent since at a duration of 15 s, the formation of AgCu bimetallic particles could not be confirmed by EDS analysis (Figure S14b). Note that in a previous work, it was shown that Cu electrodeposited as Cu^+^, and this may also explain the presence of Cu_2_O on the ED catalysts.^41^

The electrical resistivities of Ag and Cu are very low, 1.6 and 1.68 μΩ cm at 20 °C,^58^ respectively, though the combination of Ag and Cu modifies the resistivity.^39^ Moreover, the electrical resistance of complex foams and interconnected bimetallic particles may be higher due to tiny, interconnected areas among particles and the contact resistance of particles to the substrate. Herein, to get insights into these aspects, ERT experiments were performed for the first time in this kind of electrocatalyst. Note that dimensionless means that conductivity values are given.^59^ Namely, the experiments were performed by adjusting the conductivity of the water solution where the electrode was immersed so that the immersion of an electrode, made of the bare Cu foam, did not appreciably change the conductivity inside the probe. In this way, the mean value of the dimensionless conductivity with the Cu foam was close to 1. The test with Ag foam supplied a dimensionless conductivity equal to 1.85, confirming the higher conductivity of Ag in comparison with Cu. Instead, the values of 1.41 and 1.80 were measured during the test with Ag/Cu ED and Ag/Cu GD, respectively. Thus, the ERT measurements confirm that the deposition of Ag particles on Cu foam induces an increment of the conductivity of the electrode. The increment for Ag/Cu ED is, however, lower than that estimated using the Ag/Cu atomic ratio, probably because of the presence of a tiny interconnected area between the AgCu particles and the substrate that induces the presence of a non-negligible electrical resistance or the presence of copper oxide. The measured value for Ag/Cu GD is very close to that measured for the Ag foam; this result is most likely due to the high Ag loading coating that the foam copper surface obtained with the galvanic displacement.

Hence, regardless of the preparation method, the as-prepared catalysts could be described as a Cu metallic foam support coated in some regions by a Cu_2_O film, wherein bimetallic AgCu particles are deposited and highly stabilized. Note that bimetallic particles are Cu- and Ag-enriched surfaces in the electrodeposited and displaced catalysts, respectively. This configuration enhances the electron transfer between the support and the active species (vide also EIS data) and may explain the stability of the film during the electrocatalytic tests.

### Electrochemical Characterization

The electroactive surface areas of Ag/Cu displaced and electrodeposited electrodes were calculated by Pb UPD and are shown in Table 1. These EASA values are very close (∼80 cm^2^) and higher than those of the Cu and Ag foams (see below double capacitance values obtained by EIS). Note that both Ag and Cu contribute to Pb UPD.^32^ However, if the EASA is referred to the mass of Ag, the obtained values, i.e., 79 and 18 cm^2^/mg_Ag_ for ED and GD fresh samples, respectively, strongly evidence that a lower amount of Ag is available for the electrochemical reaction in dendrites than in nanoparticles.^60^ The larger particle size in the displaced sample may explain the differences.

**Table 1:** Electroactive Surface Area (EASA) Values Obtained by Pb UPD and Equivalent Electric Parameters Obtained from EIS Analysis

sample	EASA^a^ (cm^2^)	*R*_s_ (Ω cm^2^)	*R*_ct_ (Ω cm^2^)	*C*_dl_ (mF cm^–2^)	*R*_d_ (Ω cm^2^)	τ (ms)
Ag/Cu ED	79	1.9	2.7	5.79		15.7
Ag/Cu GD	81	2.3	3.1	6.57		20.4
Cu bare	15	2.0	144.3	0.21	110.6	30.3
Ag bare	29	2.8	136.9	0.24	38.2	33.2

a Ag polished plate (experimental): 3.61 × 10^–6^ C cm^–2^. Cu polished plate (experimental): 3.00 × 10^–6^ C/cm^–2^

**Figure 7:**
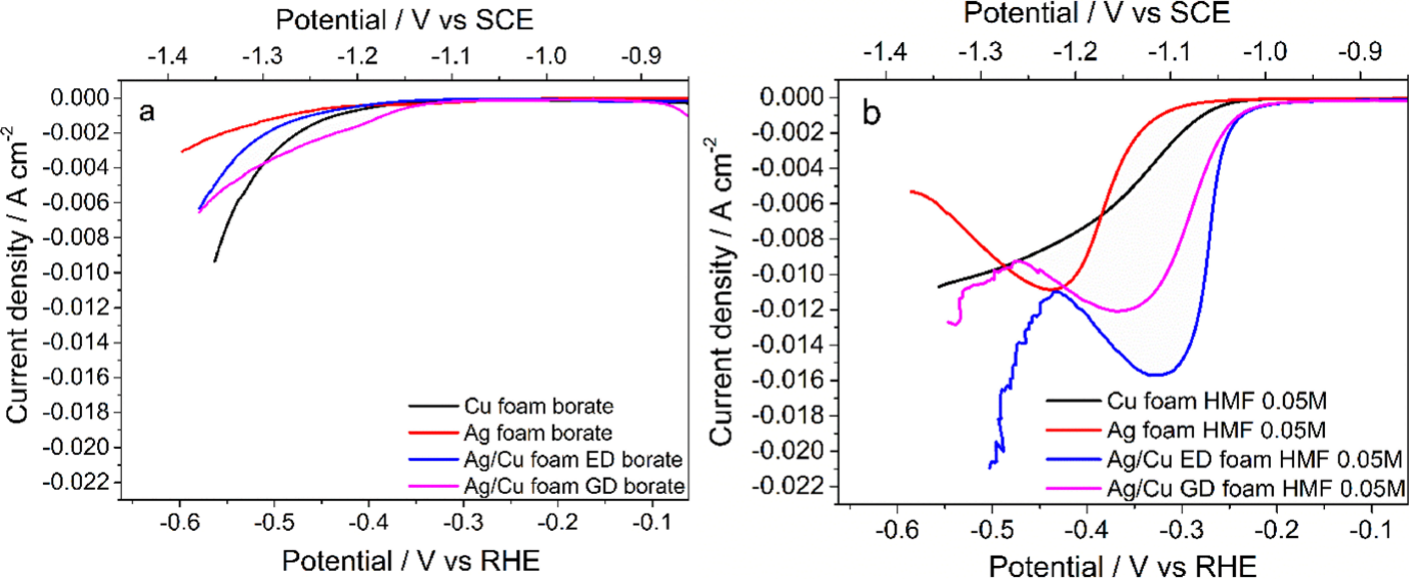
LSV curves in borate (a) and borate plus HMF (0.05 M) (b) at Ag/Cu ED, Ag/Cu GD, and Ag and Cu bare foams. The i*R*_u_ compensation was made post-analysis. Range: 0 to −1.4 V vs SCE. Scan rate: 1 mV s^–1^ for borate solutions and 5 mV s^–1^ for HMF-containing solutions.

The activity of the electrocatalysts in the aqueous blank electrolyte (borate buffer pH = 9.2) is first investigated (Figure 7a). Cu foam is more active than Ag foam, reaching a higher current density at lower overpotentials; however, both are moderate H_2_ evolution catalysts. The deposition of Ag by both ED and GD provokes a shift of the onset toward lower overpotentials with respect to the bulk Ag sample, whilst only for the displaced catalysts, the HER starts at less cathodic potentials than for Cu foam. Tafel slopes for the Ag/Cu ED and GD samples, summarized in Table 2, are very similar (95–100 mV dec^–1^), meaning similar electron transfer kinetics for both of them, albeit at lower overpotentials for the displaced catalyst. These values are around 1.2 and 2.1 times lower than those for Cu and Ag bare foams, respectively, confirming an enhanced HER kinetics on the Ag/Cu electrocatalyst surfaces. Hence, these results indicate that the nanostructuration of the foam surface with AgCu bimetallic particles by both GD and ED increases the activity in the HER in comparison with pure Ag, most probably due to the combination of Cu and Ag on the surface of the electrocatalyst and to the higher electroactive surface area.

**Table 2:** Comparison of Tafel Plot Slopes Determined in the Electrolytes Containing Sole Borate and Borate plus HMF (0.05 M)^a^

sample	slope borate (mV dec^–1^)	slope HMF (0.05 M) (mV dec^–1^)
Ag/Cu ED fresh	98.6	39.4
Ag/Cu ED spent	114.8	45.4
Ag/Cu GD fresh	95.0	56.1
Ag/Cu GD spent	107.6	61.0
Cu bare	119.4	54.6
Ag bare	195.7	73.8

a The calculations were made in the LSVs after i*R*_u_ correction.

Upon addition of 0.05 M HMF to the blank electrolyte, the characteristic HMF reduction wave appears at lower overpotentials than the HER in Figure 7b. The reduction starts at around −1.03 V vs SCE (−0.24 V vs RHE) for both ED and GD catalysts, which is ca. 100 and 50 mV lower than for Ag and Cu bare, respectively. However, the ED sample reaches a higher current density at a lower potential than the GD catalyst over the studied potential range. The absence of the peak in the Cu catalyst in 0.05 M HMF suggests a lower activity or a mass-transfer limited process. Enhanced kinetics in HMF reduction in comparison with the HER is demonstrated by a decrease in the Tafel slope for all the catalysts (see Table 2). The slope values indicate that the charge transfer occurs faster for Cu than Ag (55 vs 74 mV dec^–1^) and that only the ED method contributes to an enhancement of the kinetics in comparison with Cu foam (40 mV dec^–1^). The easy reduction of HMF has been related to the delocalization of the charge in the furan ring.^5^ In this work, it is observed that the type of catalyst determines the overpotential and the kinetics, suggesting that the HMF reduction is more affected by the catalyst structure and composition than the HER. The enhancement in the activity of the Ag/Cu electrocatalysts in the HMF reduction in comparison with Ag and Cu bare foams could be related to an increase in the intrinsic activity and/or in the mass transfer, as confirmed by the EIS analyses reported below. The differences between catalysts can be related to a different extent of the frontier orbital overlap with the HMF^5^ and the morphology that in turn modify their mass transfer resistance.

**Figure 8:**
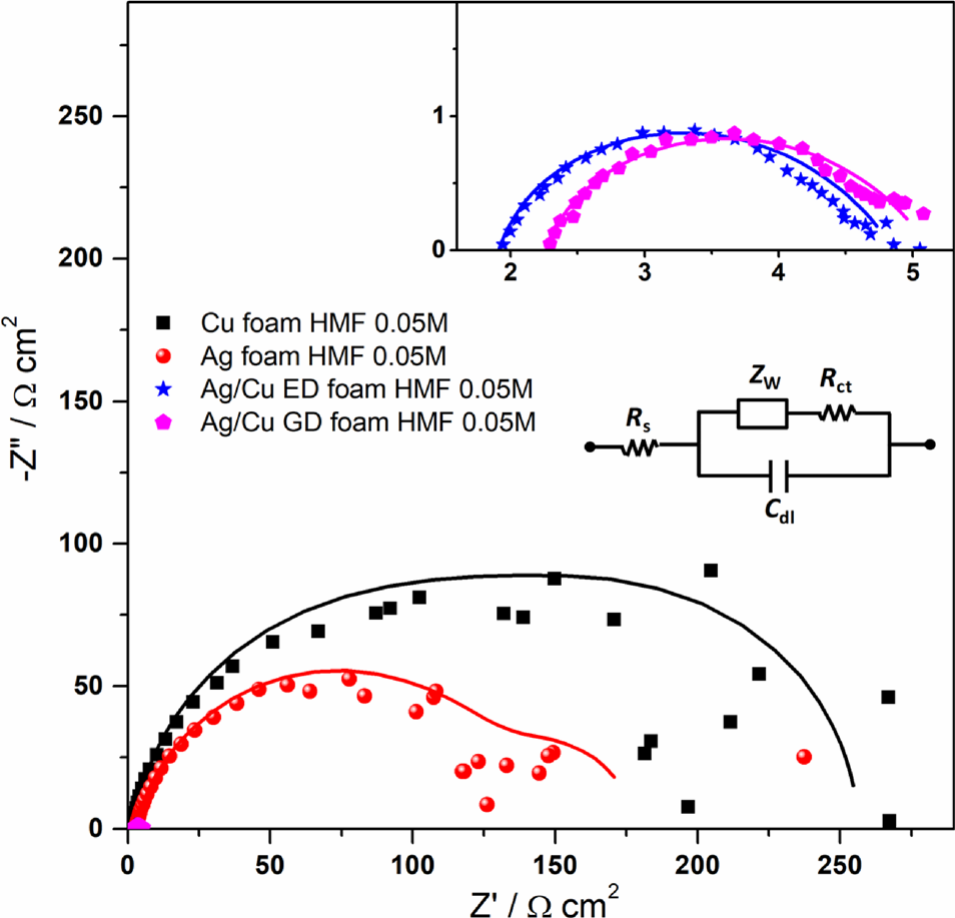
Nyquist plot from EIS measurements using the Ag/Cu ED, Ag/Cu GD, Cu bare, and Ag bare samples. The lines represent the data fitted by using the Randles equivalent circuit in the inset.

In order to shed light on the observed behavior, EIS analysis has been carried out in borate plus 0.05 M HMF solution at a −1.10 V vs SCE (−0.31 V vs RHE) potential. The results of these measurements are shown in Figure 8. It is clearly evident that the total impedance of the bare foams is much larger than those of displaced and electrodeposited samples (reported at high magnification in the inset of Figure 8). This finding is consistent with the data reported in Figure 7b, in which GD and ED catalysts are characterized by larger current densities with respect to Cu and Ag foams. Another difference among these two pairs of samples is the shape of the Nyquist plots: in fact, the bare foams exhibit a typical two-time constant spectrum constituted by two arcs (partially overlapped in the case of Cu foam), while a single arc is distinguishable in the other samples (inset of Figure 8). The experimental data are fitted through the Randles equivalent circuit shown as the inset,^61^ constituted by a series resistance *R*_s_ (accounting for electrolyte and contact resistances), a charge transfer resistance *R*_ct_, a double layer capacitance *C*_dl_, and a Warburg element *Z*_W_ (accounting for diffusion). The latter is not included in the fitting procedure of GD and ED samples since only a time constant is exhibited by these two catalysts. The obtained parameters are shown in Table 1. Similar values are obtained for *R*_s_ for all the samples, while large differences are obtained for *R*_ct_ and *C*_dl_. In particular, bare foams exhibit rather larger resistances and lower capacitances than the other two samples, implying lower intrinsic activity and EASA, in accordance with the discussion reported above. Moreover, it is noteworthy that both foams are characterized by mass transport limitation, as evidenced by the presence of the low-frequency feature in their spectra. In particular, the diffusion resistances *R*_d_, calculated from the Warburg element^62^ and reported in Table 1, confirm that this phenomenon is more pronounced in Cu foam, thus justifying the absence of a reduction peak in Figure 7b, as anticipated above. Conversely, no evidence of mass transport limitation is present in both GD and ED samples. Finally, charge transfer time constants τ are calculated by multiplying *R*_ct_ and *C*_dl_ values (see Table 1).^63^ It is found that the Ag/Cu ED sample is characterized by the lowest value followed by the Ag/Cu GD catalyst; quite larger values are exhibited by both the bare foams, implying that the charge transfer is slower for the latter samples.

By considering the results obtained from the EIS analysis, it can be confirmed that the larger activity of GD and ED samples can be attributed to their enhanced charge transfer properties and higher EASA with respect to the bare foams. In particular, the electrodeposited catalyst is characterized by the fastest charge transfer, which is responsible for the highest measured current density. Moreover, both Ag and Cu foams suffer from mass transport limitations, which further limit their electrochemical activity.

**Figure 9:**
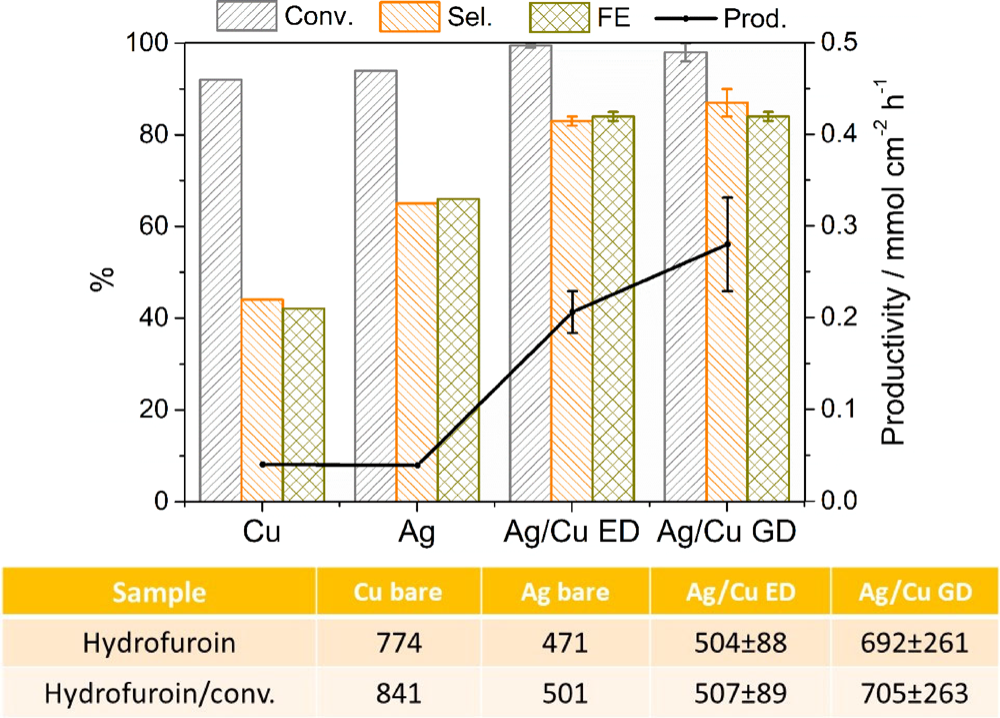
HMF conversion, selectivity to BHMF, FE, and BHMF productivity values obtained during the first electrolysis cycle at −1.3 V vs SCE in borate plus 0.05 M HMF electrolytes for Ag/Cu ED, Ag/Cu GD, and Cu and Ag bare foams. The table summarizes the areas in the HPLC chromatogram of the hydrofuroin byproduct and the areas divided by the HMF conversion.

The electroactivity of the catalysts was also investigated during electroreduction at a constant potential (−1.3 V vs SCE and −0.51 vs RHE) to consider the selectivity of the electroreduction process. Note that the electroreduction of 0.02 and 0.05 M HMF solutions is carried out over the same foam materials. In the 0.02 M HMF electrolyte, BHMF selectivities are close to 100% for both ED and GD catalysts, in agreement with data reported elsewhere;^11^ herein, the discussion is focused on the 0.05 M solution (Figure 9). Both Ag/Cu ED and GD catalysts increase the conversion of HMF (∼100%) toward BHMF in comparison with the bare foams and, in particular, the Cu electrode. Electrodeposited and displaced catalysts reach BHMF selectivity values (83 and 87%, respectively) well above those obtained for the bare foams (44 and 65% for Cu and Ag, respectively). Meanwhile, the production of the byproduct, 5,5′-di(hydroxymethyl) hydrofuroin dimer, only decreases in comparison with that of the Cu foam (table in Figure 9). Note that the dimer is generated by the coupling of radicals and its formation is fostered by increasing the HMF concentration in the electrolyte.^11^ Hence, the deposition of Ag over Cu foams also determines the selectivity of the reduction process. The conversion and selectivity in BHMF of the Ag/Cu ED and GD catalysts are rather similar after the accumulation of the same charge (241 C). However, there are remarkable differences in the BHMF productivity and specific BHMF productivity values. The BHMF productivity is higher for the GD sample since a higher current density is kept at the end of the tests and in turn the reaction time is shortened (Figure S19). On the other hand, the specific BHMF productivity, which includes the amount of Ag, is higher for the ED sample (0.290 and 0.059 mmol cm^–2^ h^–1^mg_Ag_^–1^), confirming a higher activity of this sample. Note that the productivity values are ca. 1.5–3 times higher than those for previously reported catalysts.^9,17^ It is noteworthy that long reaction times are required to perform the electroreduction with Ag and Cu bare foams due to the low exchanged current densities. Such long reaction times together with the low BHMF selectivity values lead to a drop in the BHMF productivity. A trade-off between selectivity and reaction time makes the productivity values of Ag and Cu to be similar.

The results obtained evidence that Ag/Cu catalysts are not only more active but also more selective for the reduction of the aldehyde to the alcohol than the monometallic counterparts. The trend in the activity and selectivity could be related to the electroactive surface area, the mass and charge transfer (as confirmed above by EIS), and the composition and shape of the catalyst particles, i.e., mono- and bimetallic. In an attempt to separate both contributions, the nanostructuration of the surface of the Cu and Ag bare foams is performed by electrodepositing Cu and Ag nanoparticles, respectively (see Figure S20). An enhancement in the performance is observed in Figure S21 with respect to bare foams, but the activity and selectivity became lower with respect to bimetallic samples. These results suggest that there is a synergistic role due to the presence of Ag and Cu that drives the reaction to the desired products. The configuration of the HMF molecule activated on the catalyst surface could be modified as well as the kinetics of HER and HMF reduction. Making a comparison between Ag/Cu ED and GD, the former sample is more active, showing a higher specific BHMF productivity, while the GD sample due to the hierarchical porosity may increase the mass transfer, therefore the number of aldehydes at the electrode–electrolyte interface and in turn the productivity. However, some changes in the catalyst morphology and composition could also contribute to the differences.

The stability of GD and ED samples has been evaluated by performing three cycles of reaction for each catalyst (Figures S22 and S23). Conversion, selectivity, and FE are rather constant, while the slight modifications in the productivity are related to differences in the reaction time. Tafel slopes in Table 2 reveal that the kinetics of charge transfer of the catalysts for both HER and HMF reduction decrease after the cycles. Moreover, the mass transfer could be modified because of changes in the morphology of the coatings. It is noteworthy that when a catalyst is tested directly in the reduction of a 0.05 M HMF solution (Figure S22c), the productivity is higher than that reported in Figure S22b in the first cycle, while similar values are reached in the third cycle. These results indicate that the catalyst is modified under electrochemical tests, though it reaches some kind of leveling. To further shed light on the stability of the electrocatalysts, they were deeply characterized after the electrocatalytic cycles (ca. 12 h).

### Characterization of Electrocatalysts after Tests

**Figure 10:**
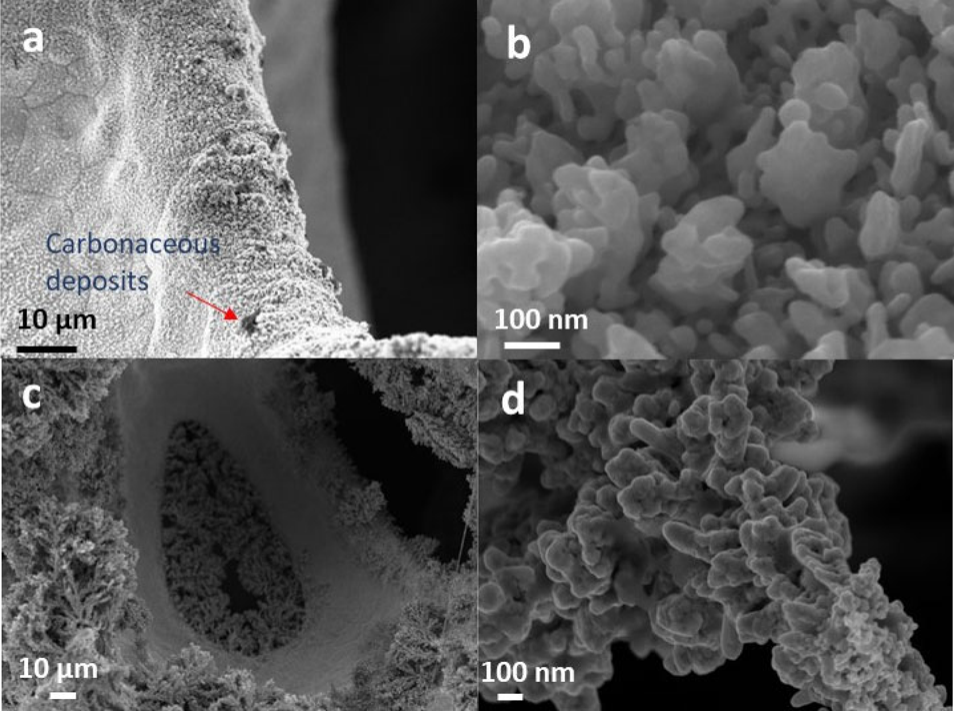
FE-SEM images of Ag/Cu ED (a, b) and Ag/Cu GD (c, d) spent catalysts. (b) and (d) are the high magnification images of the nanoparticles and a dendrite, respectively.

**Figure 11:**
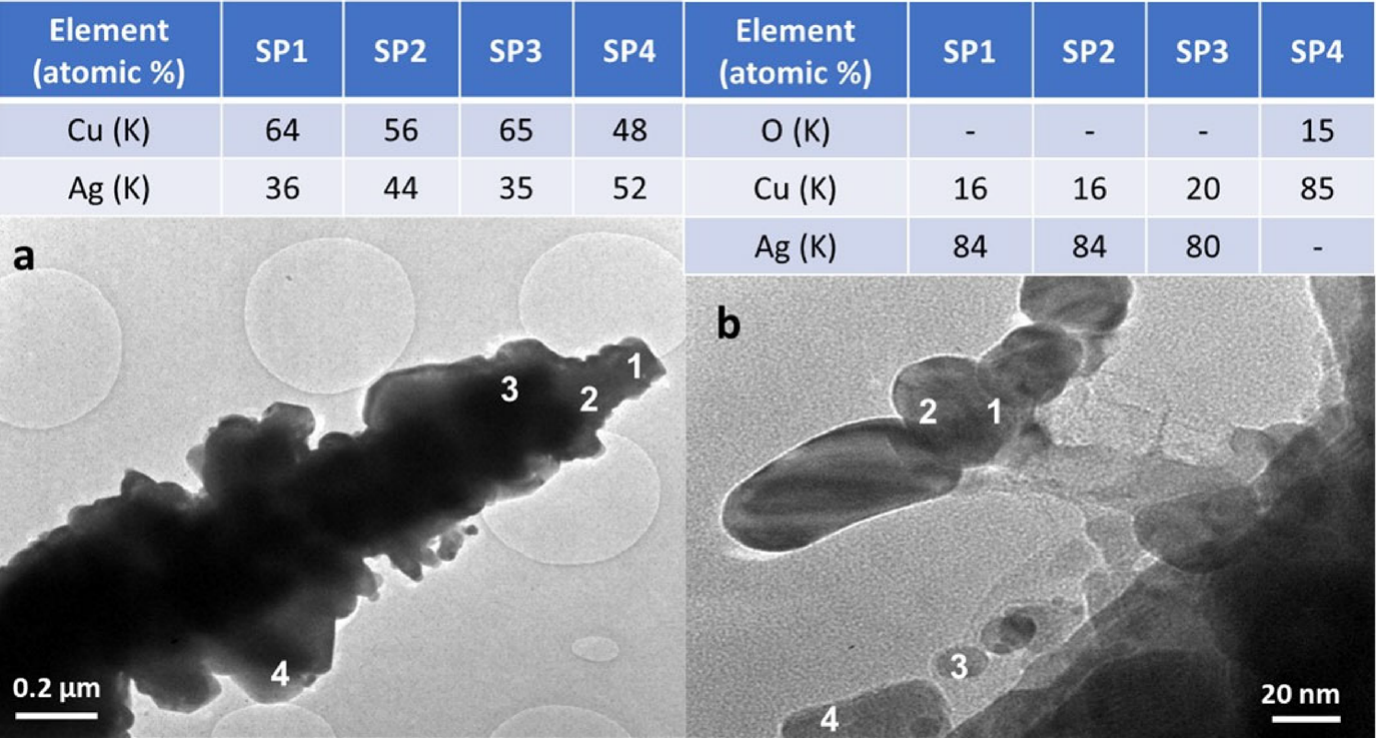
HRTEM images of the Ag/Cu ED spent catalyst: dendrite (a) and supported particles (b). The EDS data obtained in the regions of interest indicated by the numbers are summarized in the tables above the figures.

After prolonged electroreduction tests and three reaction cycles (including LSVs in blank and HMF-containing electrolytes), the coatings are rather stable in terms of adhesion (Figure 10a,c and Figure S24a). However, high-resolution FE-SEM and HRTEM images show that Ag/Cu particles are sintered in the electrodeposited and galvanic displaced spent catalysts. Indeed, the EASA of the Ag/Cu ED catalyst decreases from 79 to 45 cm^2^. After the electrochemical tests, more rounded and interconnected particles are observed in the arrays of particles and within the dendrites (Figure 10b,d, Figure S24b, and Figure 11), suggesting that the sintering occurs by both coalescence and Ostwald ripening mechanisms. Moreover, FE-SEM images show tiny metallic particles on the surface of the aggregates (Figure S25), as previously observed for Ag particles deposited by galvanic displacement.^60^ Some carbon deposits are identified in SEM images as black dots (Figure 10a) as well as by EDS analyses (Figure S24c). The polymerization of some organic compounds, such as the dimer byproduct, may be responsible for the carbon build-up on the surface of the electrode.^12^ Note that the catalysts were carefully washed with an organic solvent after reaction to try to remove the organic adsorbed compounds.

The sintering of silver particles is confirmed by XRD for the GD sample (Figure 3), which displays more intense Ag^0^ reflections in the pattern after tests. Conversely, there are no remarkable differences in the intensity and broadness of the Ag^0^ reflections before and after tests for the ED sample. For both types of samples, the reflections of Cu_2_O are not observed in the patterns of the spent samples, despite the fact that the catalysts were exposed to the air. The characterization of the Ag/Cu ED catalyst after the first LSV in borate also shows that the Cu_2_O reflections already disappear after the pretreatment (Figure S26). These results confirm the assumption that Cu_2_O identified in the fresh catalysts is generated during the deposition rather than by exposure to the air. Though the latter will contribute to the oxidation of copper (see XPS below), the Cu_2_O amount is lower than that in the fresh catalysts.

EDS/STEM analyses performed on different regions of both ED and GD spent samples (Figure 11 and Figure S27) evidence a Cu enrichment in the composition of dendrites and some supported particles (75/25 or 55/45 vs 85/15), albeit this behavior is not homogeneous. Namely, it seems that the composition of big and rounded particles remains unaltered. The Cu enrichment is also observed in the catalyst after the LSV in borate.

XPS measurements of the electrodeposited sample after reaction confirm the stability of Ag and the presence of Cu metallic and oxide species (Figure 6b). The VB spectra are composed of two different contributions, Cu 3d and Ag 4d, respectively, like in the fresh catalyst. However, these measurements reveal an enriched surface with more copper atoms after the electrochemical reaction due to the segregation of the copper from the bulk to the surface. The spectra were collected at 500 eV kinetics energy, which proves a thickness of around ∼0.9 nm. The Cu 2p and Ag 3d spectra corresponding to each sample are shown in Figure S7.

Hence, the sintering and the Cu enrichment of AgCu particles may be responsible for the differences in activity during electrocatalytic cycles. The Cu dissolution and reprecipitation^64^ and/or its migration, previously observed during O_2_ and CO_2_ reduction processes,^65−67^ could occur. The dissolution mechanism is supported by ICP analyses of the electrolytic solutions after reaction and after the LSV in borate since Cu is detected. Note that Ag leaching is only observed by ICP analysis for the GD sample, and the Ag concentration in the electrolyte after tests is below 4.5 ppm. Hence, the dissolution and subsequent electrodeposition of Cu^2+^ in the solution could take place, like during the preparation of the samples by electrodeposition. The second mechanism, the migration of copper, can be understood considering the occurrence of oxidation of Cu.^28,40,68^ This oxidation may take place not only when exposed to the air but also after immersion in the basic electrolyte; some studies confirmed that Cu is readily oxidized upon removal of the cathode bias.^42^ Indeed, the degradation of a AgCu catalyst during the CO_2_RR occurs faster when the electrolysis is performed in a discontinuous fashion.^69^

The results here obtained evidence the dynamic interdependence of catalyst stability and activity under operation conditions. The sintering and the Cu enrichment of AgCu particles during the electrocatalytic reduction of HMF in basic media modify the active sites. Thus, to establish the actual effect of the Ag and Cu synergy on the electroactivity, it is mandatory to develop in situ measurements for the catalyst characterization, like for the CO_2_ electroreduction.^42,70^ Only in this way, an accurate structure/activity relationship could be established, which will pave the way for the development of advanced electrocatalysts for the selective conversion of HMF and in general of biomass-derived compounds.

## Conclusions

The electrocatalytic activity of nanostructured Ag/Cu open-cell foam catalysts for the conversion of HMF to BHMF in basic media is related to both their morphology and composition. The as-prepared catalysts, regardless of the deposition method (electrodeposition or galvanic displacement), are made of bimetallic AgCu (not alloyed) nanoparticles, rather than monometallic Ag particles, forming a coating highly interacting with the Cu support; though the differences in mass transfer during electrodeposition and galvanic displacement lead to the formation of nanoparticles or dendrites, respectively. These catalytic structures not only increase the electroactive surface area in comparison with bare Ag and Cu foams and in turn the mass and charge transfer but also provide a high electrical conductivity between the coating and the support and the synergic activity of well-dispersed Ag and Cu active sites. Consequently, the kinetics in the HER and HMF reduction reactions and the selectivity in the conversion of the aldehyde to the alcohol during long electrolysis are increased. Remarkably, a higher Ag loading and the hierarchical porosity of dendrites in galvanic displaced samples do not provide any large improvement neither in the electrochemical properties nor in the selective conversion of HMF to BHMF in comparison with the electrodeposited sample (showing a higher charge transfer), though a higher productivity is achieved during the electroreduction process at a constant potential due to shorter reaction times. The stability of the electrocatalytic activity of both electrodeposited and galvanic displaced samples is demonstrated by a three-cycle reaction. Indeed, after electroreduction, the coating is well adhered; however, the sintering of the nanoparticles and most importantly the enrichment of the catalyst surface in Cu occur. These results indicate that the system is modified during reduction conditions in basic media and in turn the identification of the real active species is not an easy task. Hence, to establish an electrocatalytic activity/structure relationship in the reduction of HMF in basic media and in general during the reduction of biomass-derived compounds, in situ characterization techniques are needed.

## Supporting Information Available

SEM images, Raman spectra, HRTEM/STEM/HAADF/EDS information, XPS, evolution of current density during electroreduction, electroreduction results, and diffraction patterns (PDF)
