# Supplementary material for: AgCu Bimetallic Electrocatalysts for the Reduction of Biomass-Derived Compounds
Source: ACS Appl Mater Interfaces. 2021 May 11;13(20):23675–88. doi: 10.1021/acsami.1c02896 (PMC8289175; doi:10.1021/acsami.1c02896)
Supplement: Supplementary file 1 — am1c02896_si_001.pdf [file am1c02896_si_001.pdf]

Supporting Information

AgCu bimetallic electrocatalysts for the reduction of biomass derived compounds

Giancosimo Sanghez de Luna,[‖] *Phuoc H. Ho,*[‖] *Adriano Sacco, Simelys Hernández*[§,†] *Juan J. Velasco Velez,*[&] *Francesca Ospitali,*[‖] *Alessandro Paglianti,*[‡] *Stefania Albonetti,*[‖] *Giuseppe Fornasari,*[‖] *Patricia Benito*[‖,]*

[‖] Department of Industrial Chemistry "Toso Montanari, Università di Bologna, Viale Risorgimento 4, 40136, Bologna, Italy

[†] Center for Sustainable Future Technologies @POLITO, Istituto Italiano di Tecnologia, Via Livorno 60, 10144 Turin, Italy

[§] Department of Applied Science and Technology (DISAT), Politecnico di Torino, C.so Duca degli Abruzzi 24, 10129 Turin, Italy

[‡] Department of Civil, Chemical, Environmental and Materials Engineering, Università di Bologna, via Terracini 28, 40131 Bologna, Italy

[&] Fritz-Haber-Institut der Max-Planck-Gesellschaft, Faradayweg 4-6, 14195 Berlin, Germany

*patricia.benito3@unibo.it

Preparation of electrocatalysts to investigate the particle growth mechanism

Ag electrodeposition on Cu foams was performed in a single-compartment three-electrode cell controlled by a potentiostat/galvanostat Metrohm Autolab PGSTAT204, equipped with NOVA software. Foams were the working electrodes (WE), while a saturated calomel electrode (SCE) and a Pt wire were the reference electrode (RE) and counter electrode (CE), respectively. The RE was placed close to the surface of the WE in the center of the cell, while the CE was placed around them close to the walls of the cell.

The concentration of the $AgNO_3$ aqueous solution electrolyte, the deposition time and the potential applied were modified as summarized in Table S1.

Table S1. Electrodeposition conditions to investigate the particle growth mechanism on the Cu foam

$AgNO_3$ concentration / mM	Potential / vs SCE	Time / s
5	-0.9	15
5	-0.9	50
5	-0.7	25
10	-0.9	50
5	-	3
5*	-0.9	25

* no stirring

Ag galvanic displacement was performed by immersing Cu foams in a beaker containing 25 mL of $AgNO_3$ aqueous solutions. The foams were attached to a rotor and kept under mechanical stirring of 200 rpm. The concentration of the $AgNO_3$ aqueous solution and the deposition time were modified as summarized in Table S1.

Table S2. Galvanic displacement conditions to investigate the particle growth mechanism on the Cu foam

$AgNO_3$ concentration / mM	Time / min
5	2.5
5	5 + 5
10	5
10*	5

* no stirring

After synthesis, all the catalysts were rinsed gently with ethanol and water.

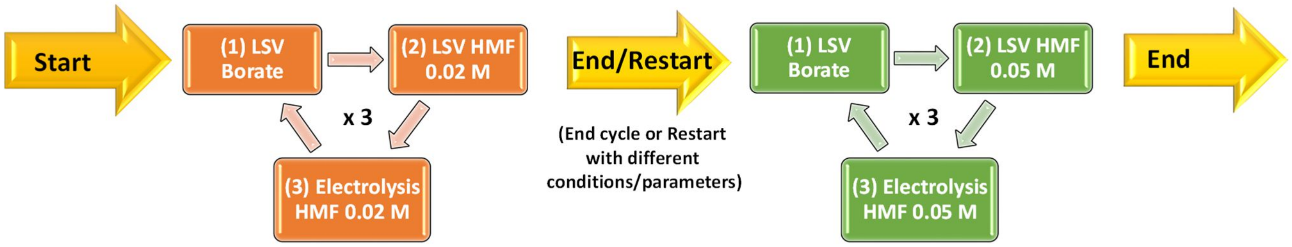

Scheme S1. Description of the catalytic cycle.

Each cycle consists of:

1. LSV in borate buffer (pH= 9.2)
2. LSV in borate plus HMF
3. Electrochemical reduction.
4. LSV in borate buffer (pH= 9.2)
5. LSV in borate plus HMF.

After the cycle, the tests could be stopped or started again with the same or different HMF concentration.

The reactions were performed collecting the theoretic charge to selectively convert all HMF in solution into BHMF. The reaction time is not fixed but depends on the type of catalyst, the catalytic cycle and HMF concentration. The duration of the electrocatalytic tests for the Ag/Cu GD and ED samples in 0.05 M HMF solution is shown in Fig. S19 and S23.

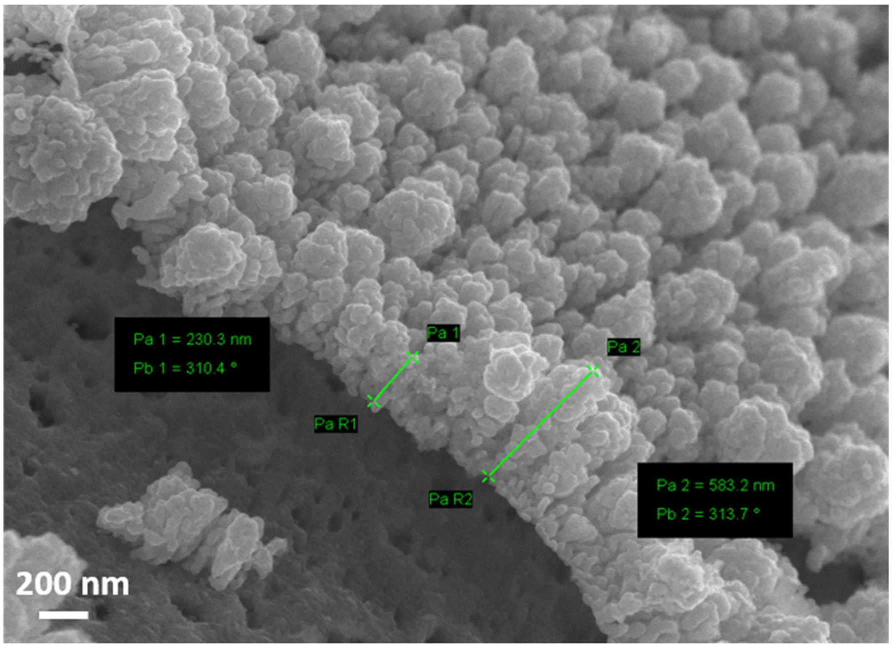

Figure S1. FE-SEM image of a cross-section of the Ag/Cu ED foam used to estimate the thickness of the coating.

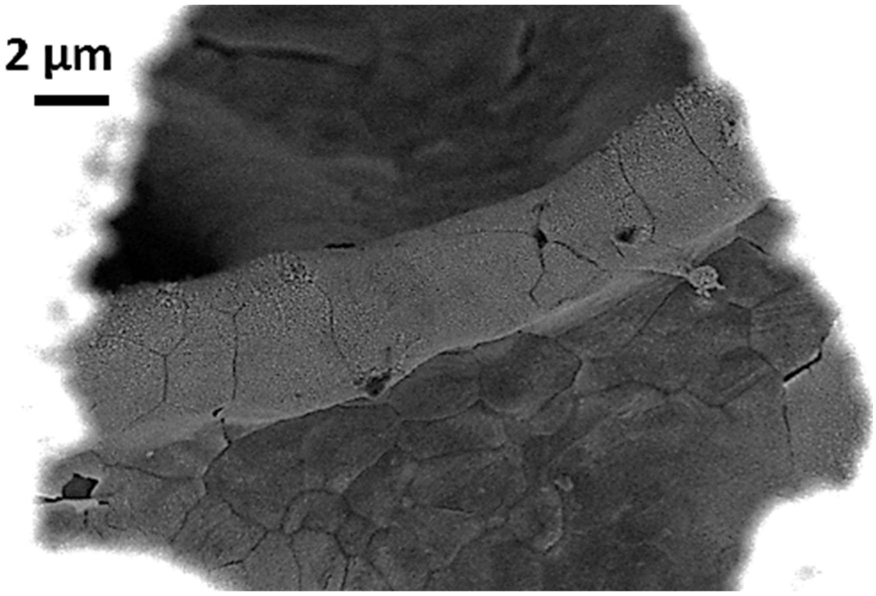

Figure S2. FE-SEM detail of a coated strut in the inner part of the Cu disk. It confirms that the deposition occurs on both the outer and inner surfaces of the foam disks

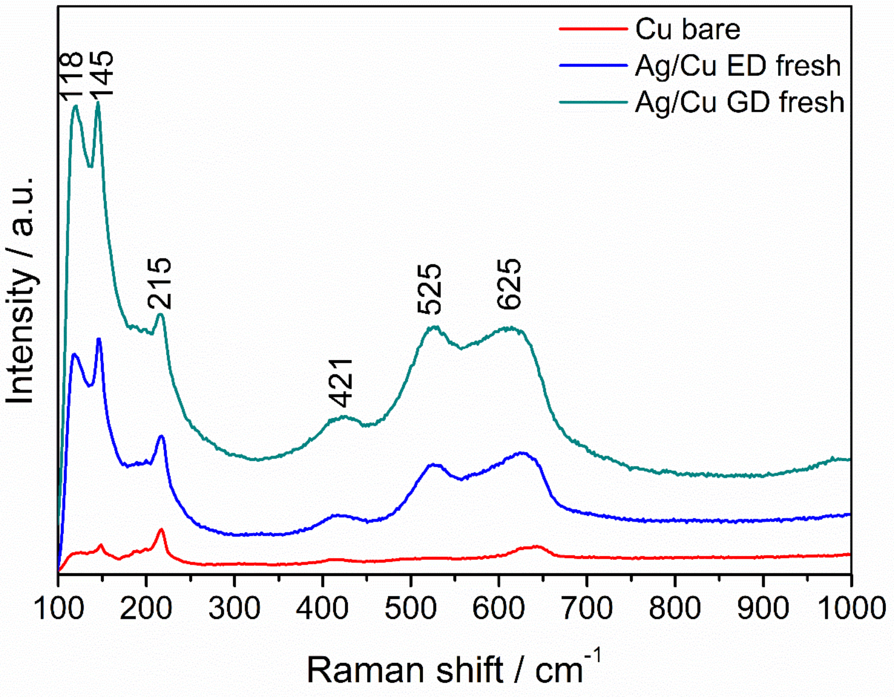

Figure S3. Raman spectra of Ag/Cu ED and GD fresh samples. For comparison purposes, the Raman of Cu bare foam is included. The Raman bands of Cu_2O at 145, 215, 421, 525, and 625 cm^{-1} are identified.

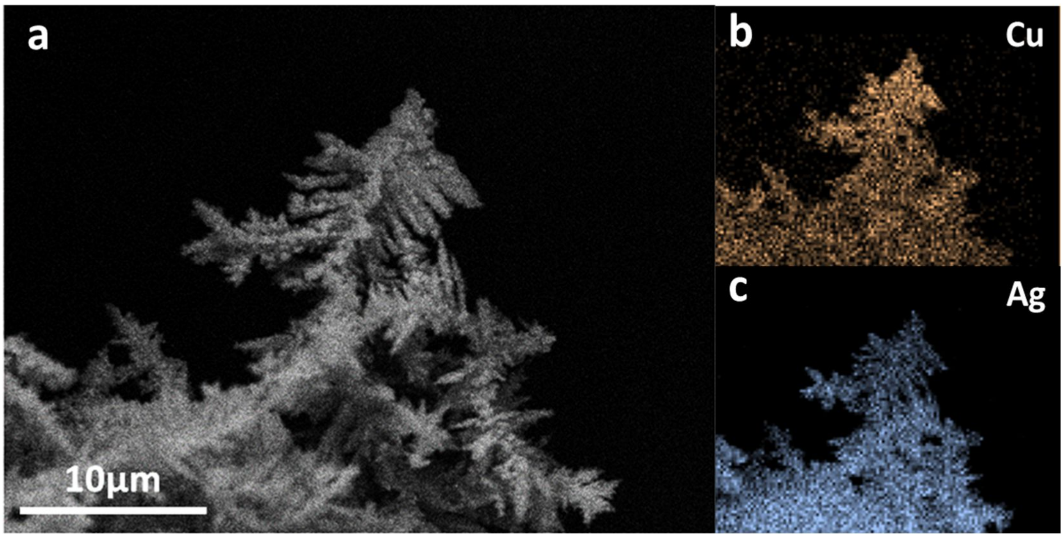

Figure S4. SEM image of a dendrite in the Ag/Cu ED sample (a) and EDS elemental maps: Cu (b) and Ag (c).

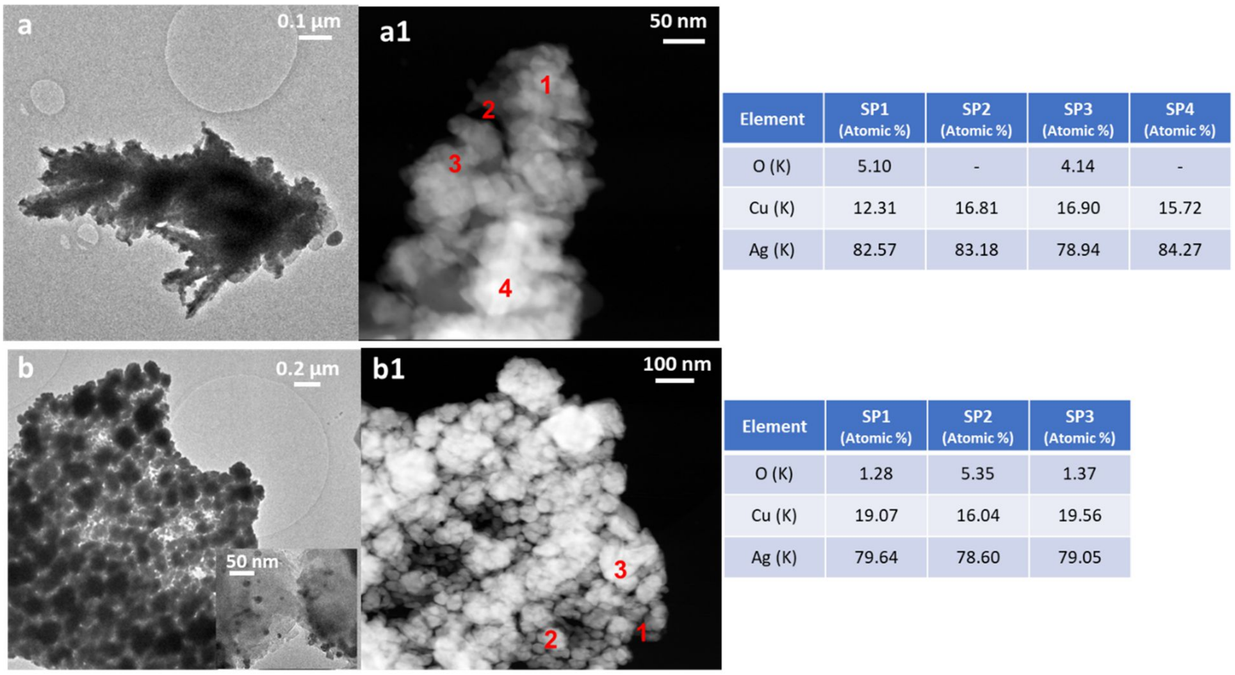

Element	SP1 (Atomic %)	SP2 (Atomic %)	SP3 (Atomic %)	SP4 (Atomic %)
O (K)	5.10	-	4.14	-
Cu (K)	12.31	16.81	16.90	15.72
Ag (K)	82.57	83.18	78.94	84.27

Element	SP1 (Atomic %)	SP2 (Atomic %)	SP3 (Atomic %)
O (K)	1.28	5.35	1.37
Cu (K)	19.07	16.04	19.56
Ag (K)	79.64	78.60	79.05

Figure S5. HRTEM and HAADF/STEM images of a dendrite (a, a1) and the coating made by arrays of particles (b, b1) in Ag/Cu GD. The EDS data obtained in the regions of interest indicated by the numbers are summarized in the tables on the right of the figures.

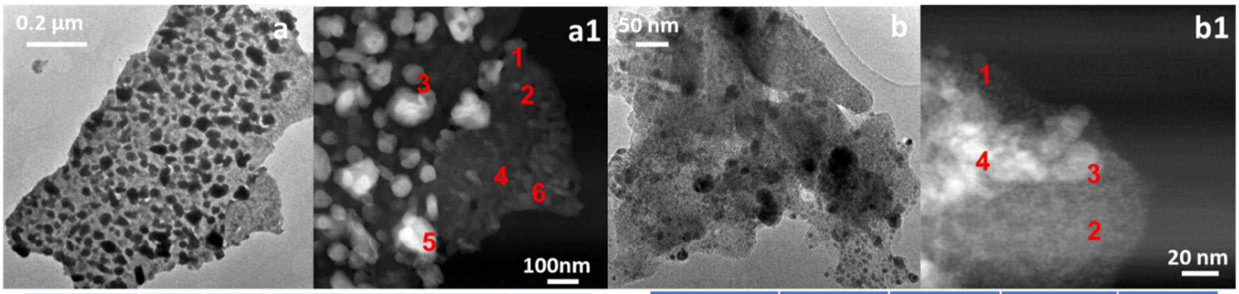

Element (Atomic %)	SP1	SP2	SP3	SP4	SP5	SP6
Cu (K)	27	20	17	17	15	21
Ag (K)	73	80	83	83	85	79

Element (Atomic %)	SP1	SP2	SP3	SP4
O (K)	67	38	40	20
Cu (K)	27	61	43	61
Ag (K)	6	1	17	19

Figure S6. HRTEM images (a, b) and HAADF/STEM images (b, b1) of the coating made by arrays of particles in Ag/Cu ED. The EDS data obtained in the regions of interest indicated by the numbers are summarized in the tables below the figures.

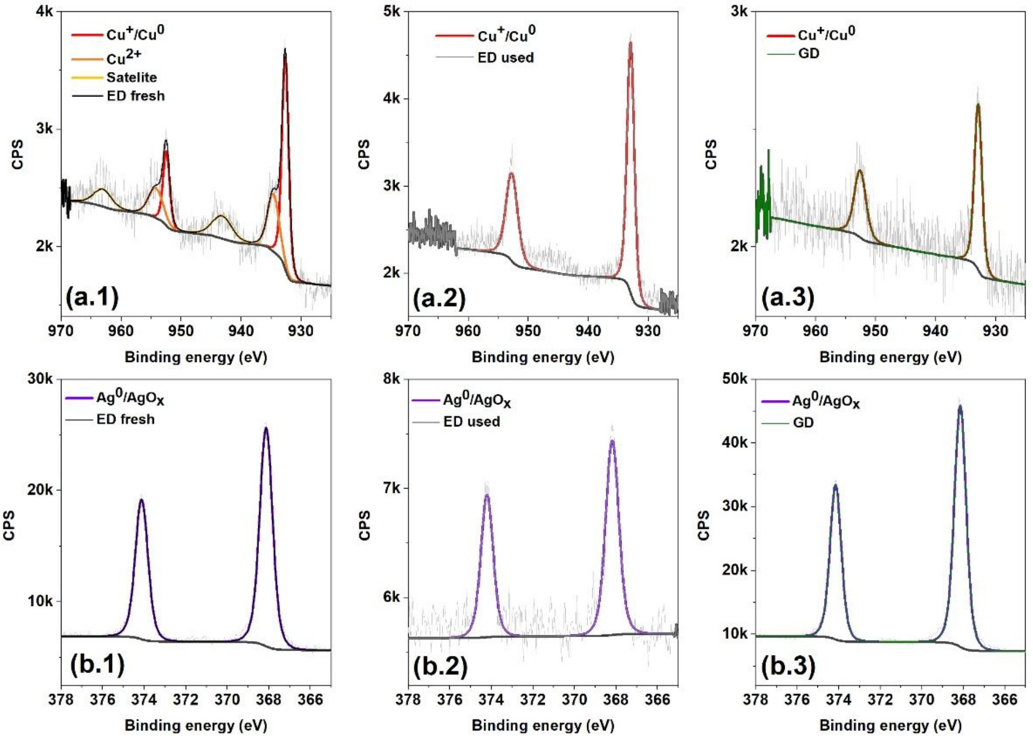

Figure S7. Cu 2*p* and Ag 3*d* XPS spectra of the Ag/Cu ED fresh (a.1, b.1) and after reaction (a.2, b.2) and Ag/Cu GD fresh (a.3, b.3)

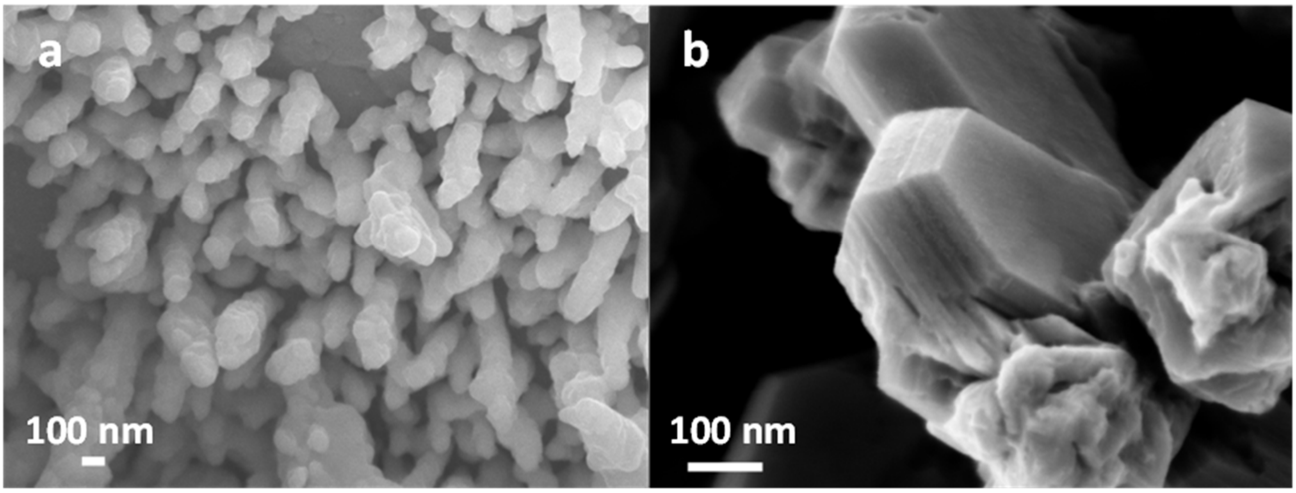

Figure S8. FE-SEM images of an Ag/Cu GD sample showing the growth of the dendrites (a) and a detail of a well-defined particle (b).

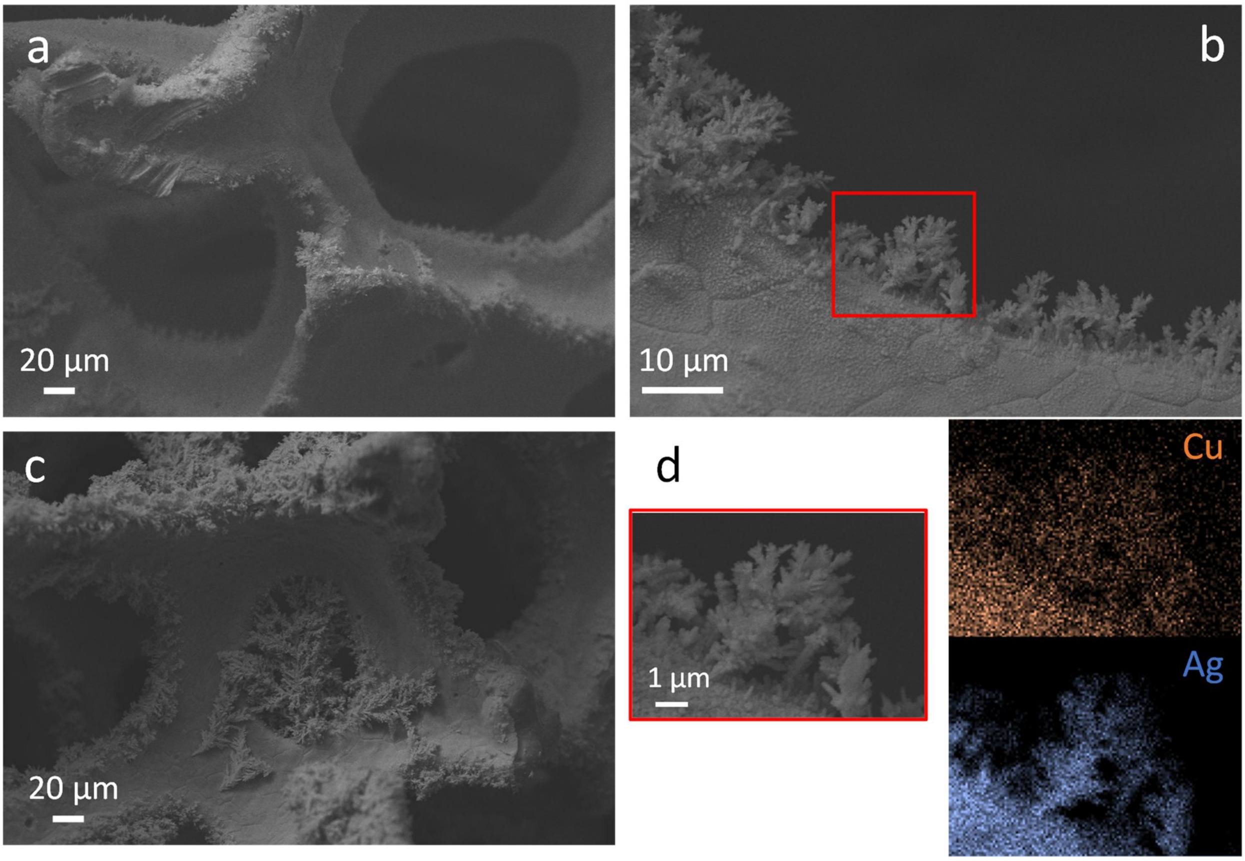

Figure S9. SEM/EDS data of an Ag/Cu foam prepared by galvanic displacement for 2.5 min with a 5 mM $AgNO_3$ solution. a and c: low magnification secondary electron images; b: detail of the short dendrites deposited on the edges of the pores; c: Cu and Ag EDS elemental maps of the region indicated by the red square in b.

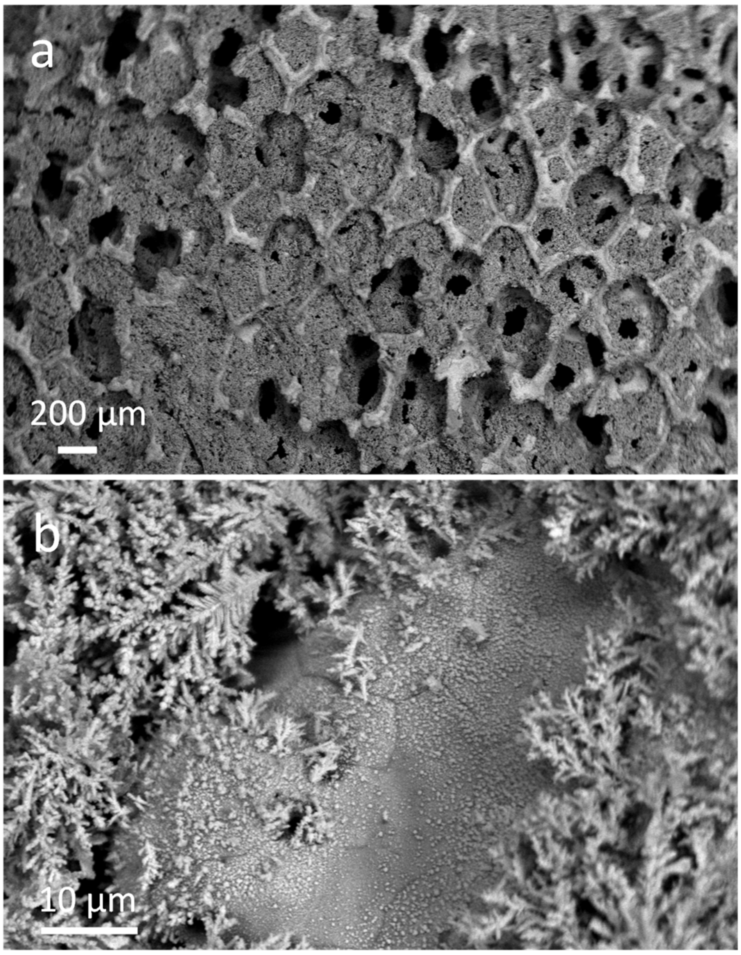

Figure S10. SEM backscattering images of a Cu foam coated twice (5 + 5 min) by galvanic displacement using the 5 mM $AgNO_3$ solution. a: overview of the pores blocked with dendrites and the struts with a lower coating; b: detail of a strut coated by the arrays of particles and the dendrites on the edge.

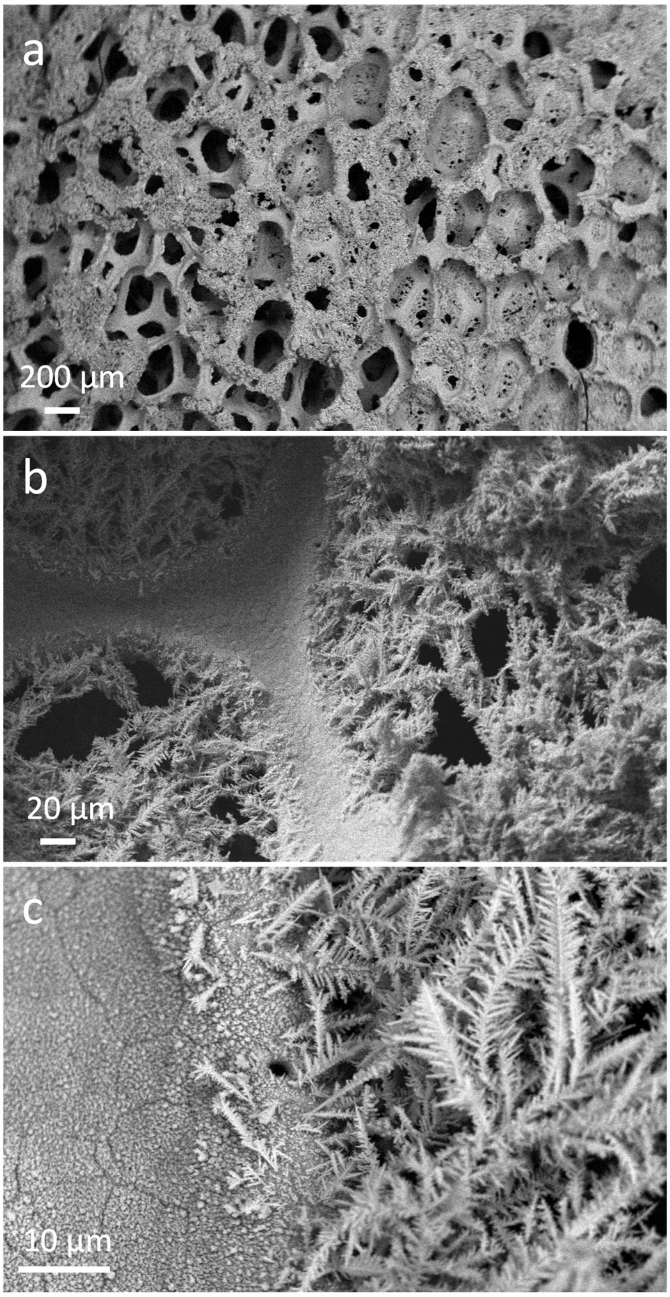

Figure S11. SEM backscattering images of a Cu foam coated by galvanic displacement for 5 min using a 10 mM $AgNO_3$ solution. a: overview of the coating distribution at low magnification; b and c: detail of a strut coated by the arrays of particles and the dendrites on the edge.

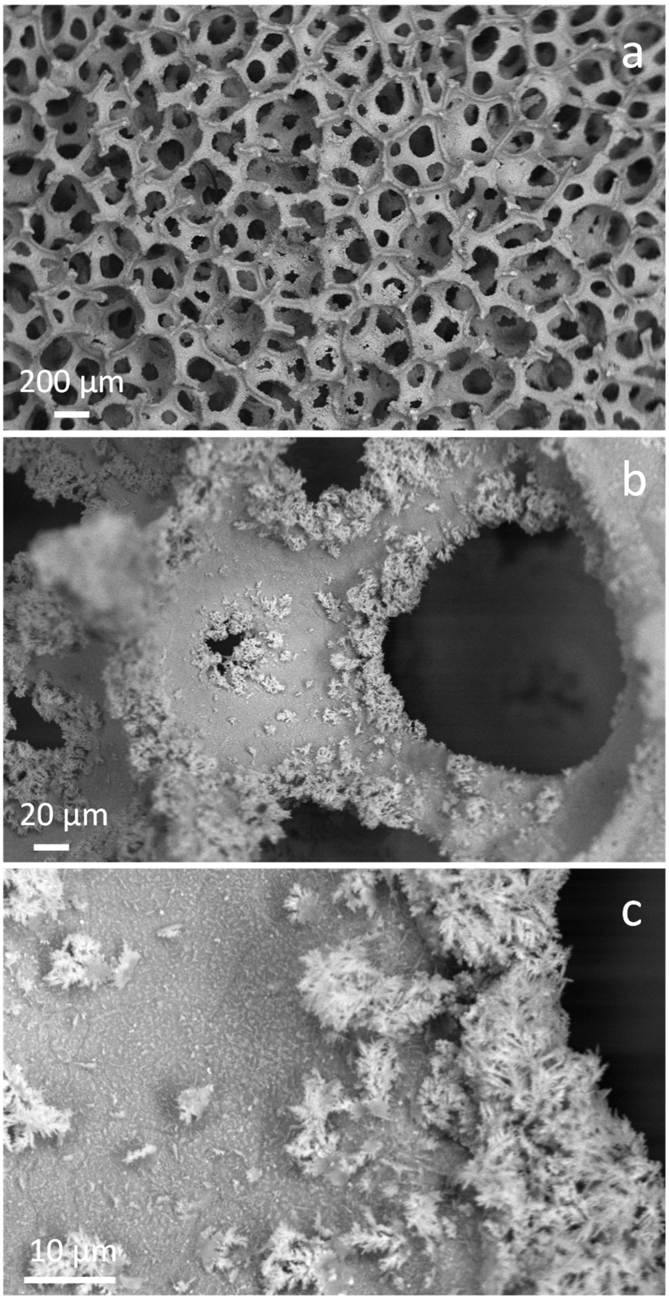

Figure S12. SEM backscattering images of a Cu foam coated by galvanic displacement for 5 min using a 10 mM $AgNO_3$ solution without stirring. a: overview of the coating distribution at low magnification; b and c: detail of a strut coated by the arrays of particles and the dendrites on the edge.

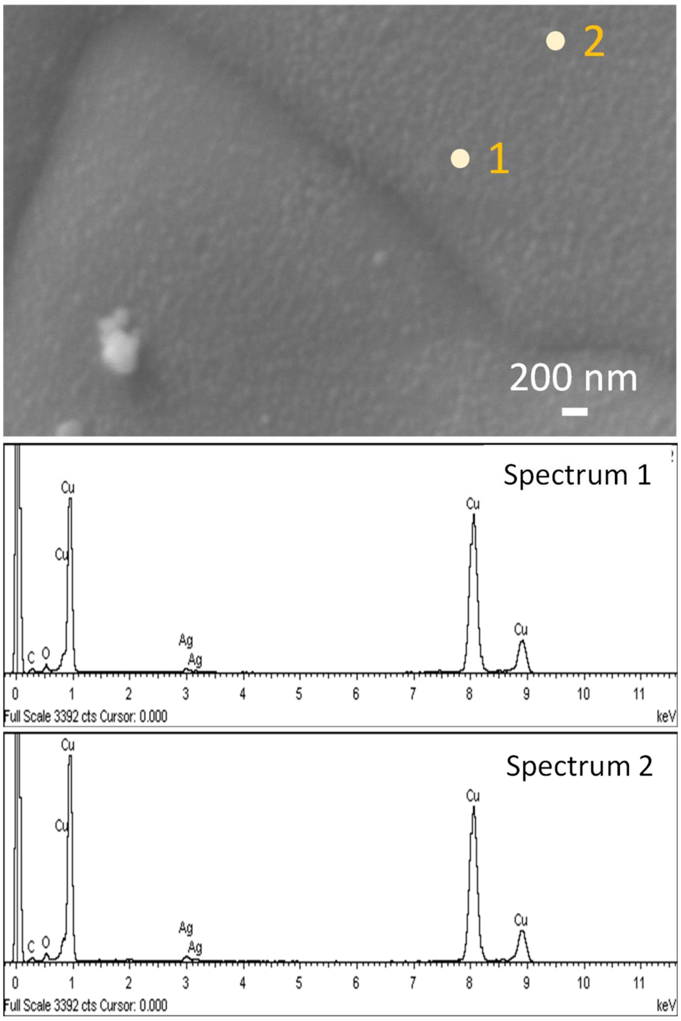

Figure S13. SEM image of a Cu foam immersed for 3 s in the 5 mM $AgNO_3$ solution. EDS of the regions labelled 1 and 2 in the image.

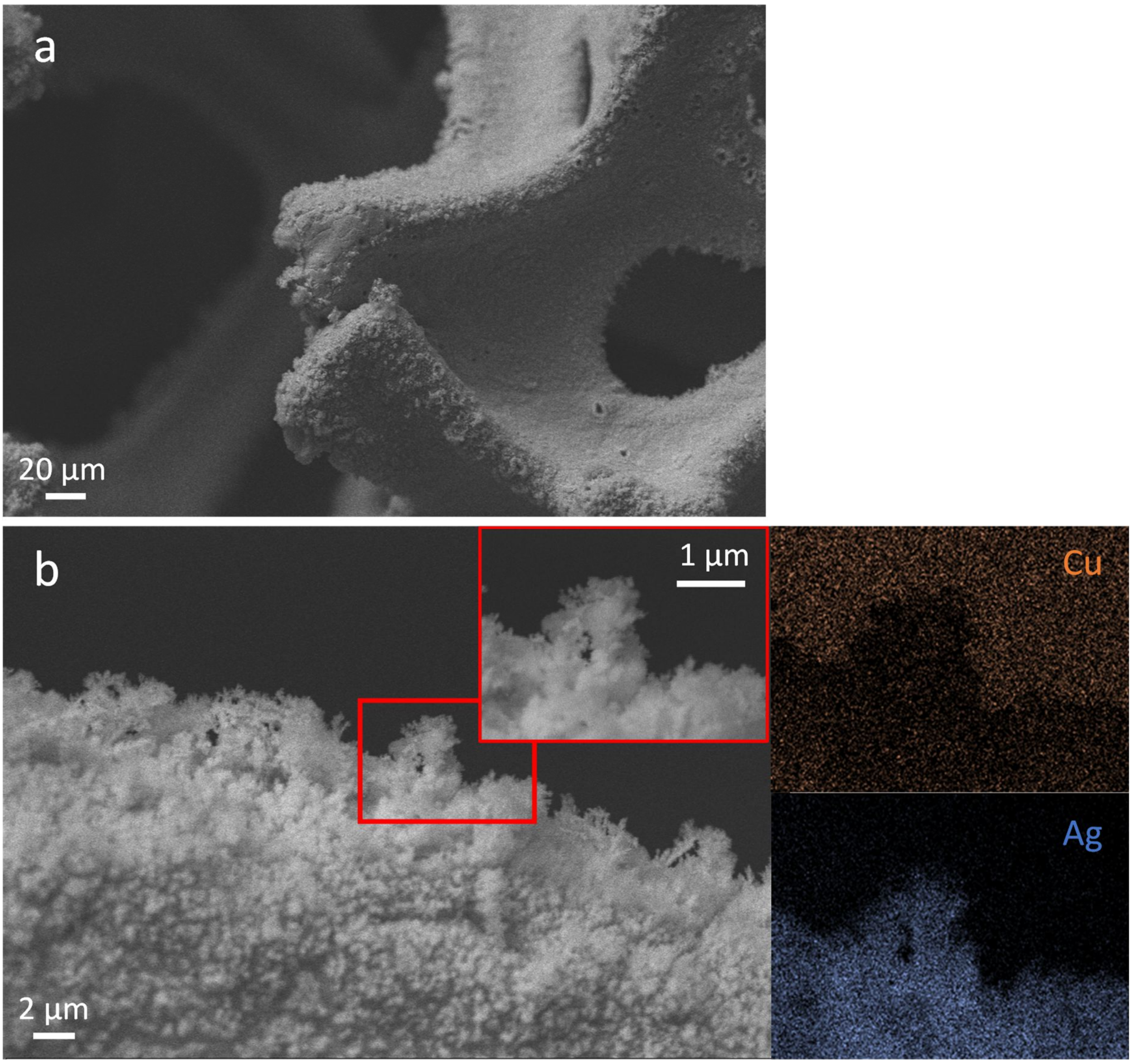

Figure S14. SEM/EDS data of an Ag/Cu foam prepared by electrodeposition at -0.9 V vs SCE for 15 s, electrolyte 5 mM $AgNO_3$ solution. a: low magnification secondary electron image; b: high magnification image showing small fractal structures and Cu and Ag EDS elemental maps of the region indicated by the red square.

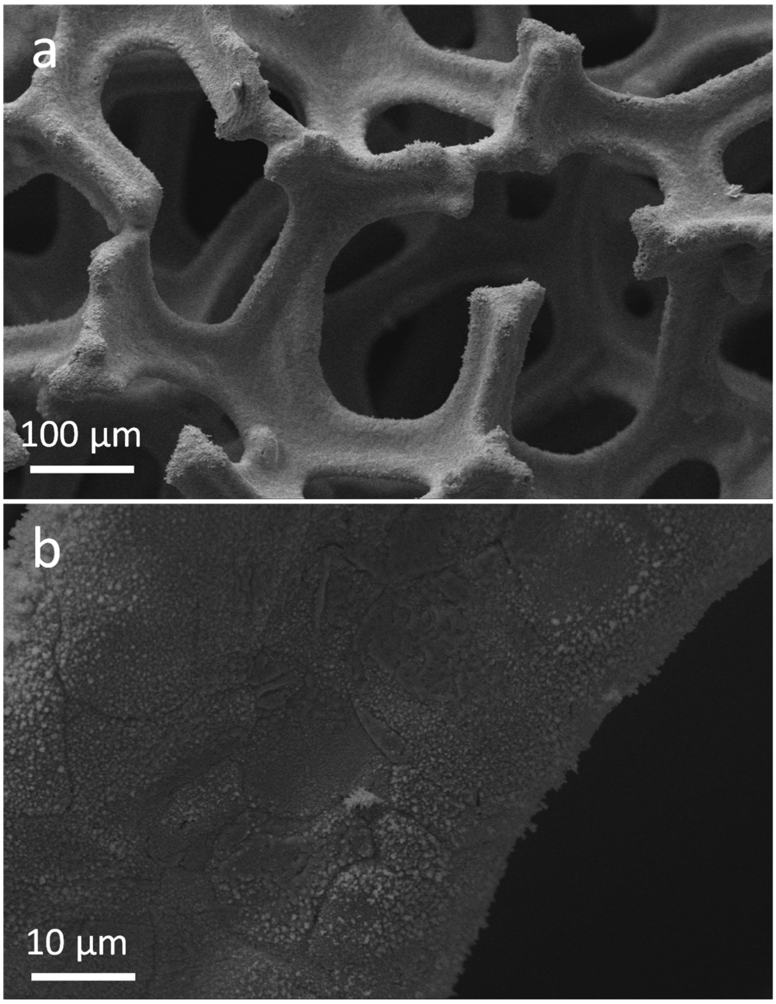

Figure S15. SEM images of an Ag/Cu foam prepared by electrodeposition at -0.7 V vs SCE for 25 s, electrolyte 5 mM $AgNO_3$ solution. a: low magnification secondary electron image; b: detail of the surface of the foam coated by aggregates of particles.

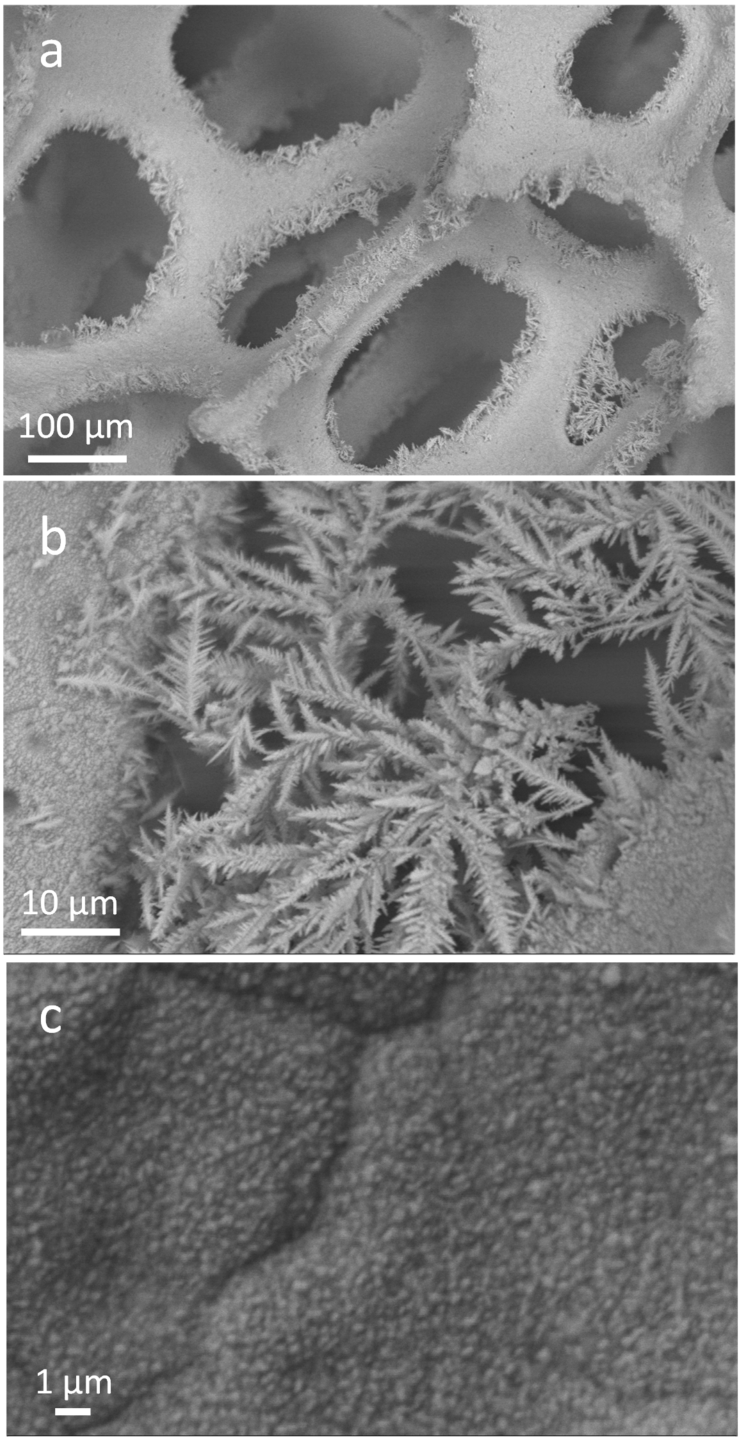

Figure S16. SEM images of an Ag/Cu foam prepared by electrodeposition at -0.9 V vs SCE for 50 s, electrolyte 5 mM $AgNO_3$ solution. a: low magnification secondary electron image; b: detail of a pore filled with dendrites; c: detail of the surface of the foam coated by aggregates of particles.

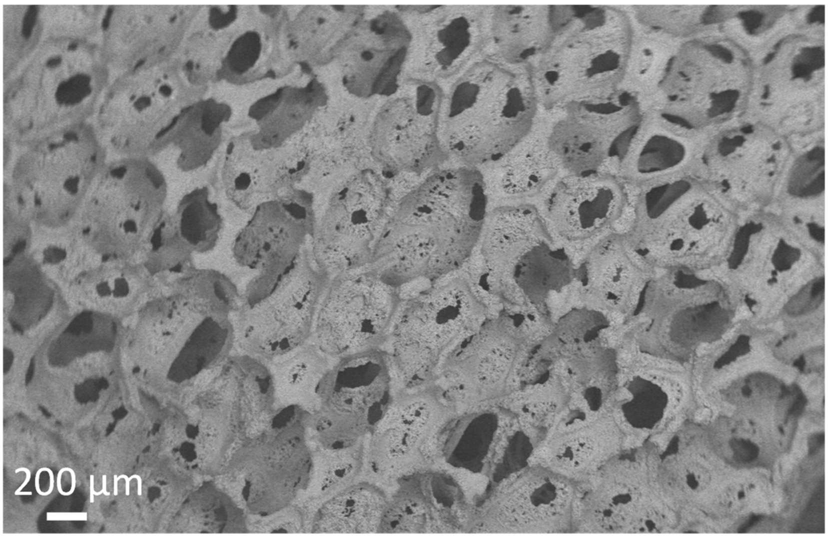

Figure S17. SEM image of an Ag/Cu foam prepared by electrodeposition at -0.9 V vs SCE for 50 s, electrolyte 10 mM $AgNO_3$ solution.

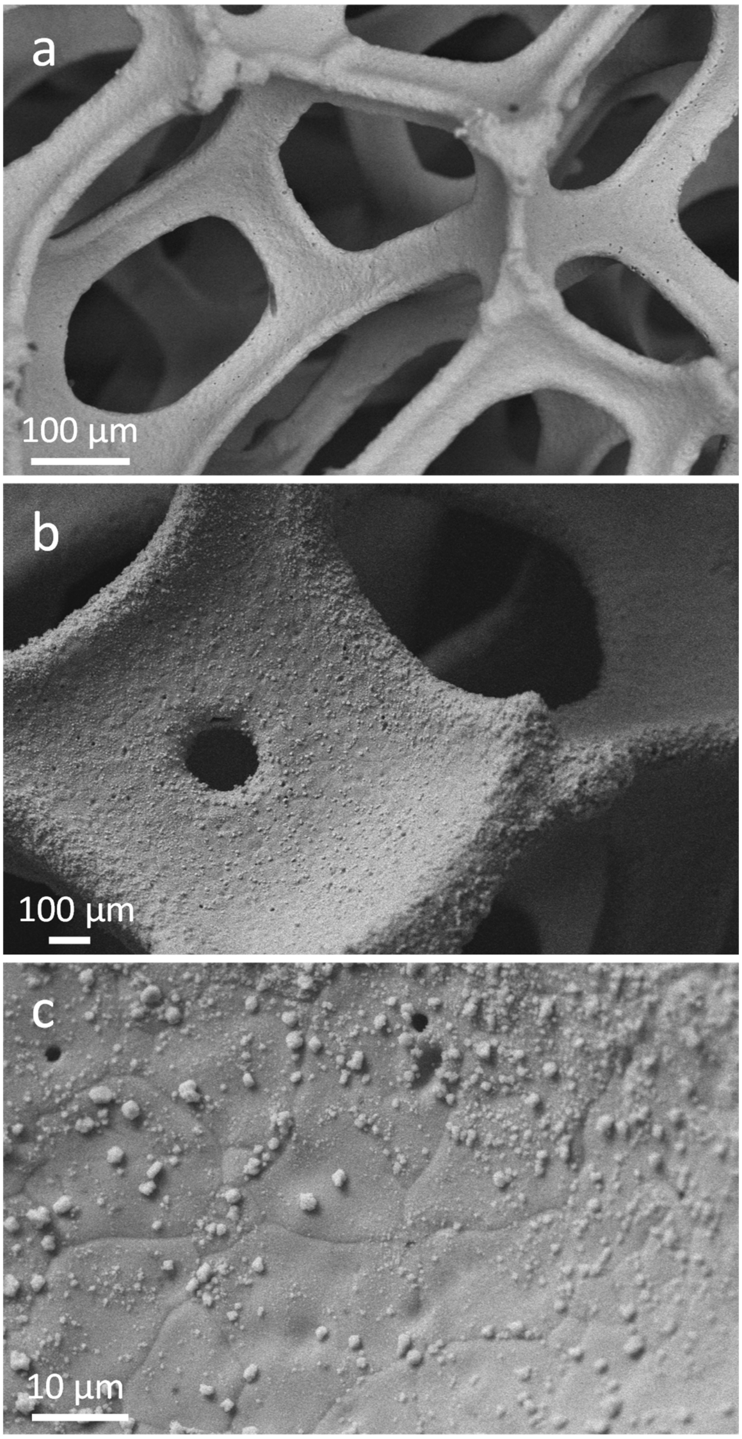

Figure S18. SEM images an Ag/Cu foam prepared by electrodeposition at -0.9 V vs SCE for 25 s, electrolyte 10 mM $AgNO_3$ solution without stirring.

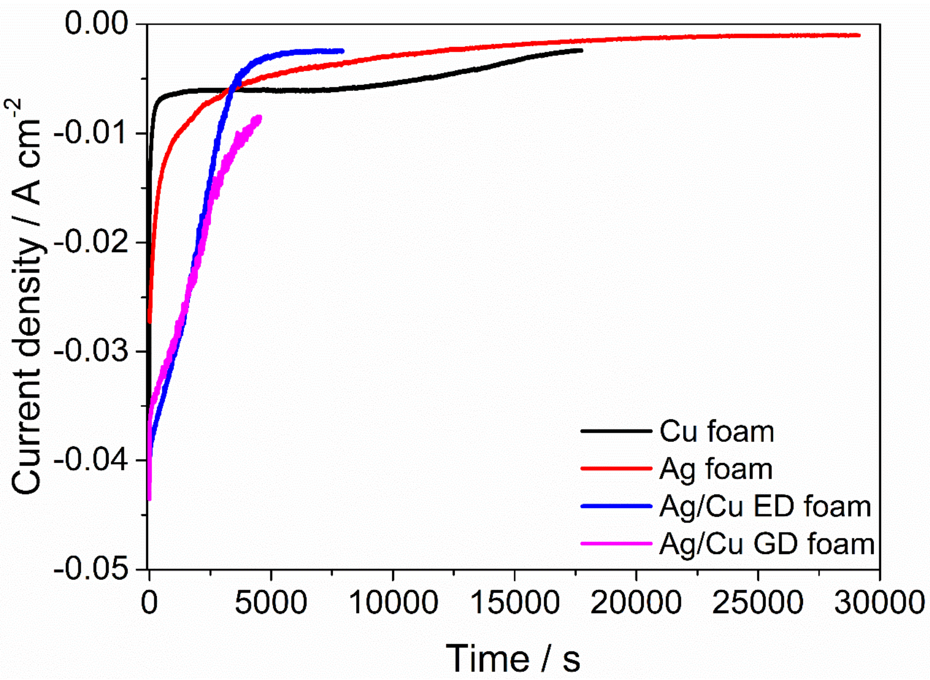

Figure S19. Evolution of the current with time during electrolysis in 0.05 M HMF in borate buffer (pH = 9.2) electrolyte at -1.3 V *vs* SCE (-0.51 *vs* RHE) over Ag and Cu bulk foams and Ag/Cu electrodeposited and displaced foams. Accumulated charge 241.23 C.

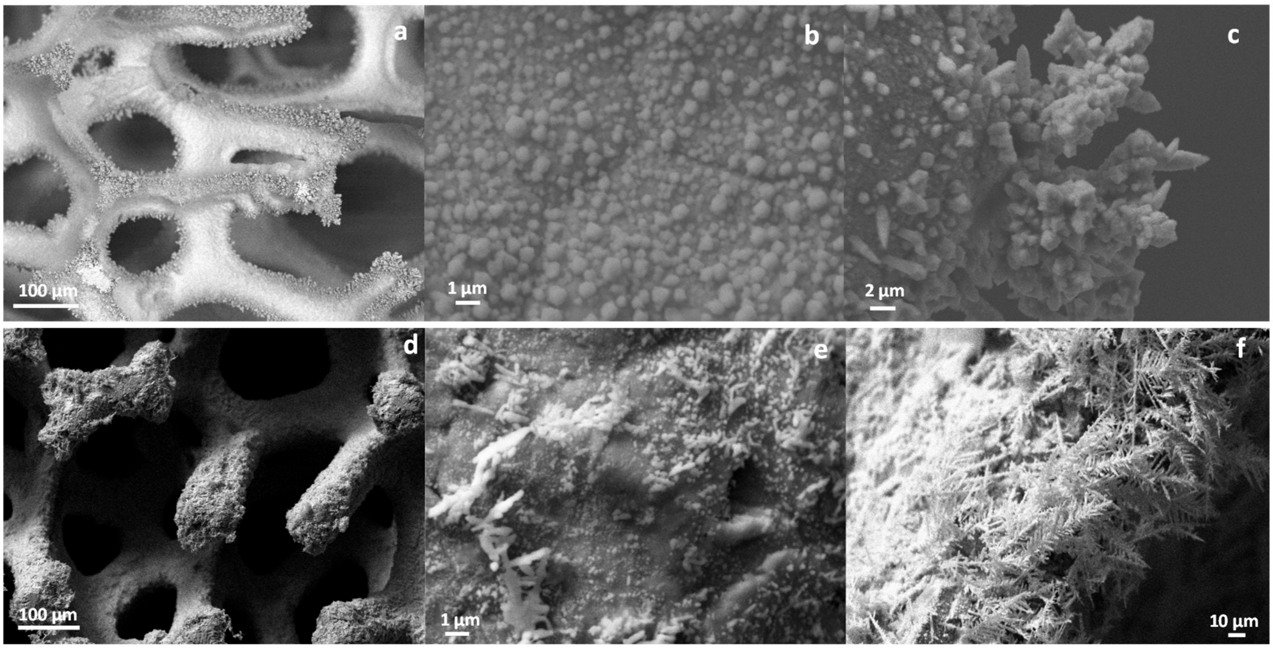

Figure S20. SEM images of Cu/Cu (a-c) and Ag/Ag (d-f) electrocatalysts. (a, d) low magnification image of the foam surface; (b, e) high magnification image of aggregates of particles; (c, f) high magnification image of dendrites.

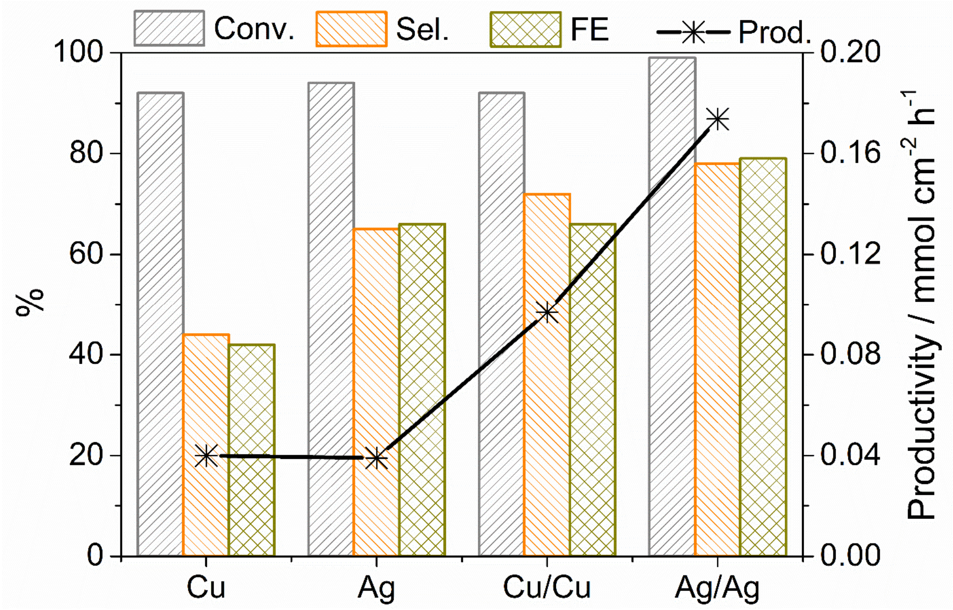

Figure S21. HMF conversion, selectivity to BHMF, FE, and BHMF productivity values obtained during electrolysis at -1.3 V vs SCE in borate plus 0.05 M HMF electrolytes for Ag/Ag, Cu/Cu, and Ag and Cu bare foams.

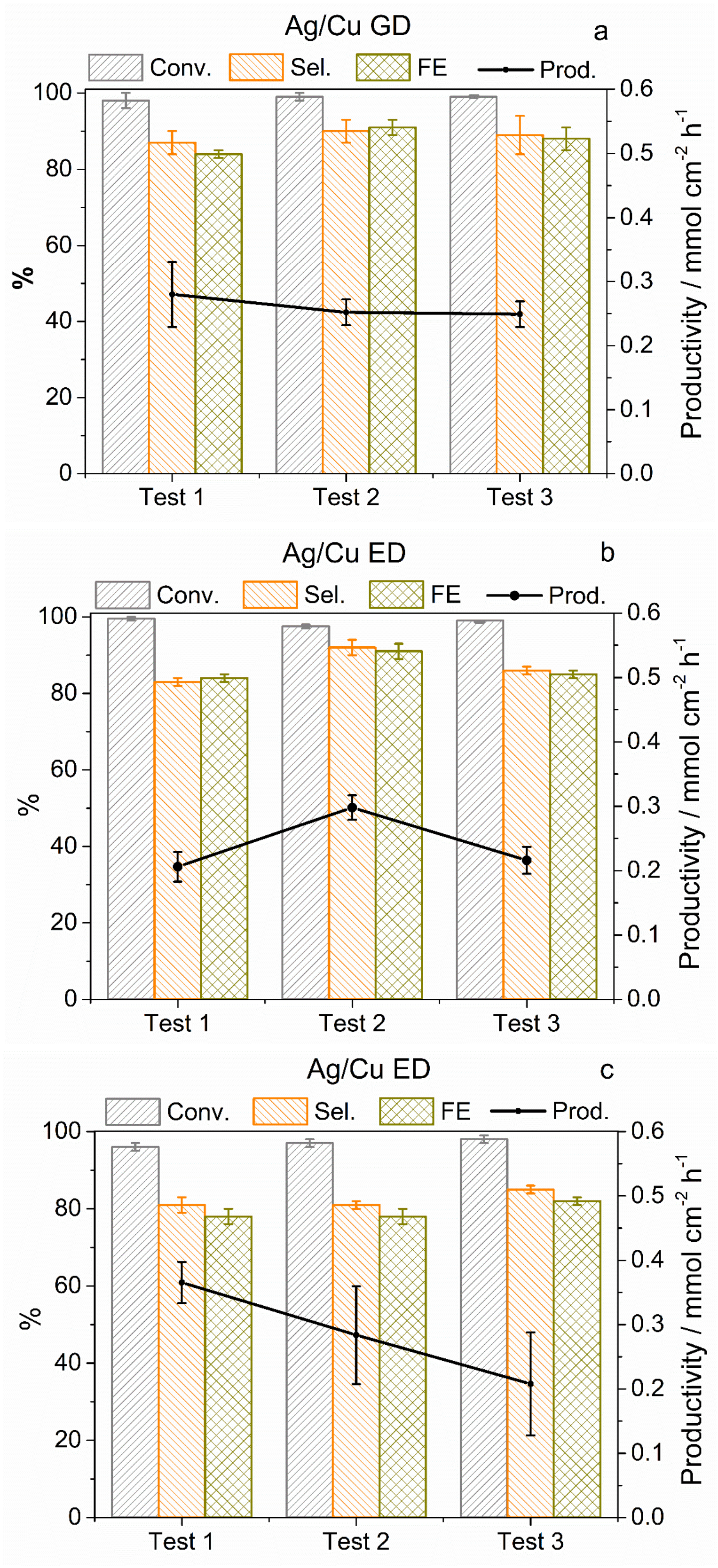

Figure S22. HMF conversion, selectivity to BHMF, FE, and BHMF productivity values obtained during electrolysis at -1.3 V *vs* SCE in borate plus 0.05 M HMF electrolytes for three consecutive electrolysis (Test 1, Test 2, and Test 3). Results obtained for: a,b) Ag/Cu GD (a) Ag/Cu ED (b) obtained for catalysts previously tested in a catalytic cycle with a 0.02 M HMF solution; c) Ag/Cu ED only tested in a 0.05 M HMF solution.

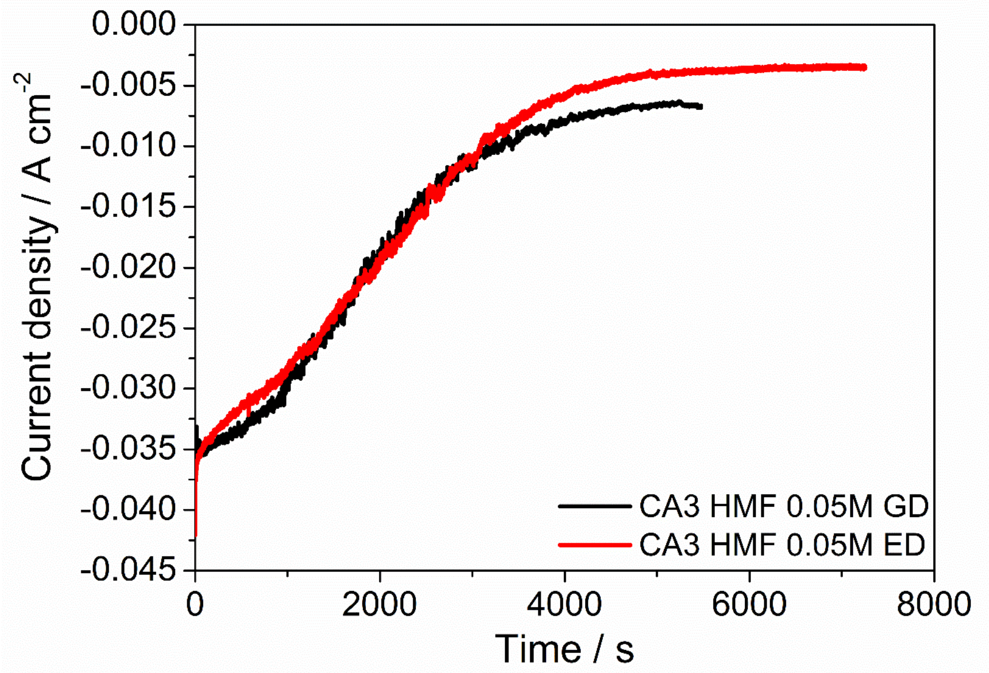

Figure S23. Current transients were recorded during the third electrolysis (CA3) in borate plus 0.05M HMF electrolyte over Ag/Cu GD and Ag/Cu ED sample.

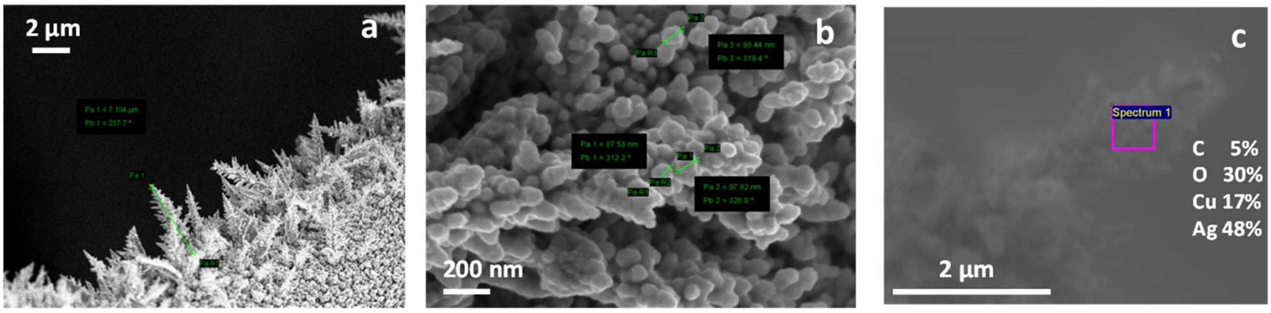

Figure S24. FE-SEM images of Ag/Cu ED spent catalyst. Low (a) and high (b) magnification images of dendrites deposited on the border of the foam surface. STEM image and EDS composition of a dendrite (c).

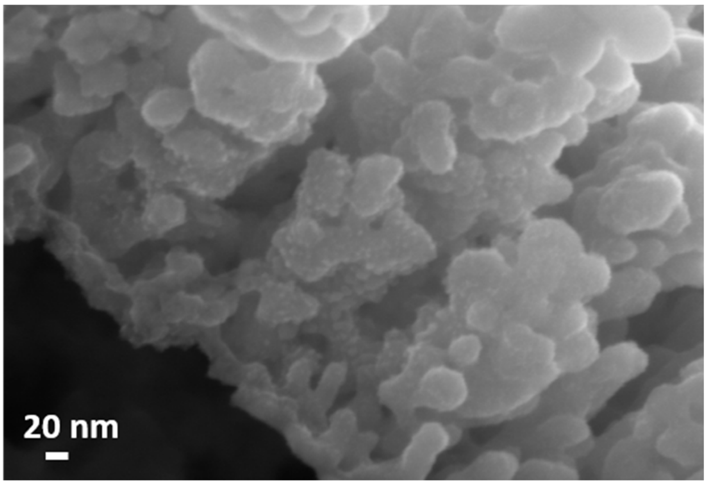

Figure S25. FE-SEM image showing the detail of the surface of an Ag/Cu ED spent catalyst.

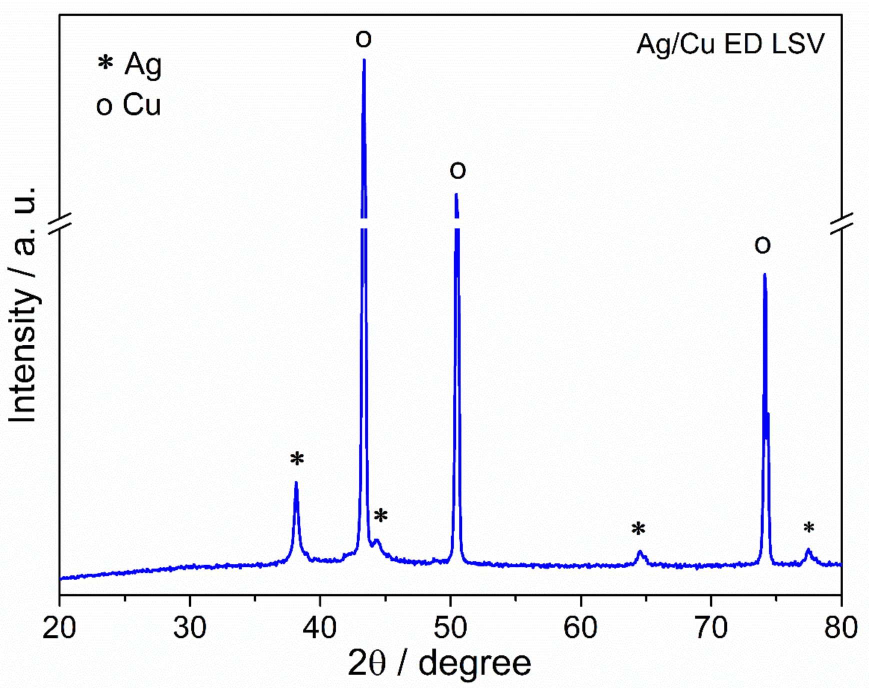

Figure S26. XRD pattern of Ag/Cu ED catalyst after the first LSV in borate buffer solution (pH 9.2).

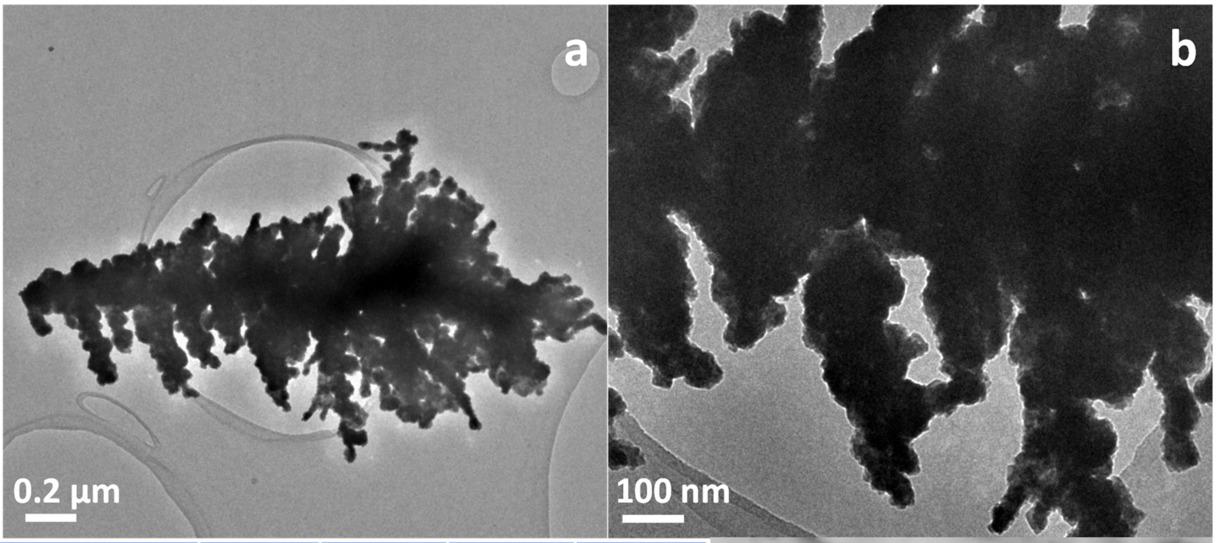

Element (atomic %)	SP1	SP2	SP3	SP4
O (K)	-	-	-	17
Cu (K)	24	26	45	36
Ag (K)	76	74	55	47

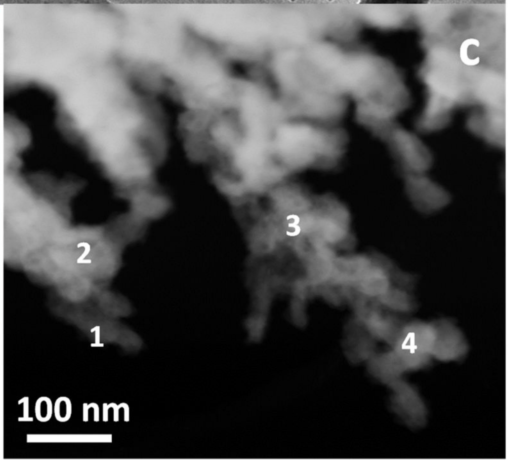

Figure S27. HRTEM (a, b) and HAADF/STEM (c) images of a dendrite in an Ag/Cu GD spent catalyst. The EDS data obtained in the regions of interest indicated by the numbers in (c) are summarized in the table.
